# Active Visual Art Therapy and Health Outcomes: A Systematic Review and Meta-Analysis

**DOI:** 10.1001/jamanetworkopen.2024.28709

**Published:** 2024-09-12

**Authors:** Ronja Joschko, Caroline Klatte, Weronika A. Grabowska, Stephanie Roll, Anne Berghöfer, Stefan N. Willich

**Affiliations:** 1 Institute of Social Medicine, Epidemiology and Health Economics, Charité – Universitätsmedizin Berlin, Berlin, Germany; 2 Formerly Charité – Universitätsmedizin Berlin, Berlin, Germany

## Abstract

**Importance:**

Art therapy has a long-standing tradition in patient treatment. As scientific interest in its use has recently grown, a comprehensive assessment of active visual art therapy is crucial to understanding its potential benefits.

**Objective:**

To assess the association of active visual art therapy with health outcomes across patient groups and comparators.

**Data Sources:**

The systematic literature search included the Cochrane Library, Embase, MEDLINE, CINAHL, ERIC, American Psychological Association PsycArticles, American Psychological Association PsycInfo, PSYNDEX, the German Clinical Trials Register, and ClinicalTrials.gov. No filters regarding language were applied. The search covered all dates before March 2021. Data analysis was conducted from April 24 to September 8, 2023.

**Study Selection:**

Randomized clinical trials with any type of patient population comparing the intervention with any control not using active visual art therapy were included. Two researchers independently screened the abstracts and full texts.

**Data Extraction and Synthesis:**

Data extraction followed the Preferred Reporting Items for Systematic Reviews and Meta-Analyses guidelines, and study quality was evaluated using the Cochrane Risk of Bias tool. Data were synthesized using narrative summaries, forest plots, and random effects meta-analyses.

**Main Outcome and Measures:**

In line with the protocol, all outcome measures of the included studies were extracted.

**Results:**

The search identified 3104 records, of which 356 outcomes of 69 studies were included, with a total of approximately 4200 participants, aged 4 to 96 years, in the review. The meta-analyses included 50 studies and 217 outcomes of 2766 participants. Treatment indications included mental, neurological, and other somatic disorders, and prevention. Most outcome measures focused on depression, anxiety, self-esteem, social adjustment, and quality of life. Art therapy was associated with an improvement in 18% of the 217 outcomes compared with the controls (1%), while 81% showed no improvement. The standardized mean difference in the change from baseline of the meta-analyses of 0.38 (95% CI, 0.26-0.51) and posttest analysis of 0.19 (95% CI, 0.12-0.26) also indicated an improvement of outcomes associated with art therapy. Overall study quality was low.

**Conclusions and Relevance:**

In this systematic review and meta-analysis of randomized clinical trials, visual art therapy was associated with therapeutic benefits for some outcomes, although most studies were of low quality. Further good-quality studies are needed to provide additional insights for its best possible integration into routine care.

## Key Points

**Question:**

Is active visual art therapy associated with patient health outcomes?

**Findings:**

In this meta-analysis including 50 studies with 217 outcomes and 2766 individuals, evidence was markedly heterogeneous regarding outcomes, population, and study quality. Based on the available evidence, active visual art therapy was associated with an improvement in 18% of the patient outcomes.

**Meaning:**

The findings of this study suggest that, given its association with patient outcomes, visual art therapy may be considered a valuable addition to standard medical care.

## Teaser

This systematic review and meta-analysis examines the use of visual art therapy in the prevention and treatment of mental health disorders.

## Introduction

The use of art therapy is widespread across many countries and disciplines,^1^ especially in the areas of mental health,^2^ rehabilitation, pain management, holistic cancer treatment, and care of older individuals. Various versions of art therapy are used in a wide variety of settings, including hospitals,^3^ schools,^4^ prisons,^5^ or nursing homes.^6^ Despite its popularity, to our knowledge, the use of art therapy was never systematically documented, which impedes the estimation of the number of global recipients. The lack of documentation and scarcity of large studies might be the reason why art therapy is not always routinely funded and integrated into standard care. Our study contributes to closing this gap.

A report from the World Health Organization has summarized the existing evidence on art and health in a scoping review, triangulating different study designs, patient groups, and indications.^7^ After including more than 900 publications in the review, the authors concluded that the arts have a great potential to enhance and maintain good health. While this extensive report has provided many useful insights into the benefits of art therapy and has further established the role art can play in gaining and maintaining health, the review has been criticized for the absence of a systematic literature search and a quality screening of the individual studies.^8^

We set out to conduct what is, to our knowledge, the first systematic review and meta-analysis of all existing randomized and controlled art therapy evidence in a single report that also takes quality of the included studies into account. We therefore believe that it may be a helpful addition to the World Health Organization report and other existing literature. To provide an overview of the complex evidence base, we aimed to gather all currently available studies on the effectiveness of art therapy. Since the overall study quality and methods are very diverse, we decided to focus on randomized clinical trials (RCTs), which are considered the standard for evaluating interventions and limit some of the bias associated with other study designs.^9^

With this systematic review and meta-analysis, we aimed to gather all available evidence regarding the effectiveness of active visual art therapy (AVAT). Since we are particularly interested in enhancing the well-being of patients, we decided to focus on the application of AVAT both to alleviate current symptoms and as a preventive measure.

## Methods

**Figure 1.**
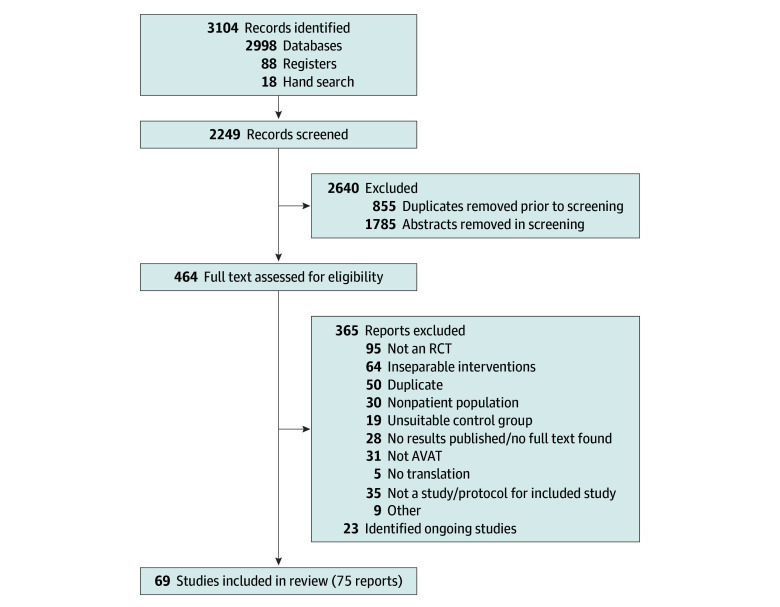
Flowchart of the Study Selection Process for the Systematic Review of AVAT The number of included studies and reports is not identical, because some articles published outcomes in separate reports, while others reported more than 1 study. AVAT indicates active visual art therapy; RCT, randomized clinical trial.

The protocol for this systematic review and meta-analysis has been registered with PROSPERO (CRD42021233272), and details of the methods have been published.^10^ We followed the Preferred Reporting Items for Systematic Reviews and Meta-Analyses (PRISMA) reporting guideline,^11^ and the Cochrane handbook.^12^ The literature search flow diagram is shown in Figure 1.

### Eligibility Criteria

#### Study Design, Intervention, and Control Group

We defined AVAT as any type of artistic activity in which patients would actively manipulate materials with their hands, such as drawing, painting, ceramic sculpting, any form of arts and crafts, and sand painting. Not included were all forms of music therapy, dance therapy, literature-based therapy, and therapy based on performing arts. To keep the diverse range of art therapies as comparable as possible, we also excluded all digital interventions.

As per our predefined search criteria, we only included RCTs in which one group used AVAT and the other group used anything other than AVAT (including no intervention, treatment as usual, attention control, or a non-AVAT intervention). Studies that used art as a diagnostic tool but not as an intervention were excluded.

#### Participants

We included all patient groups and groups receiving targeted preventive art therapy. Reasons for exclusion were induced moods (eg, artificial anxiety induction in healthy intervention groups), studies on healthy college students, or educational classroom settings with no clear preventive or curative target.

#### Outcomes

We included all RCT outcomes in our review. To identify the range of conditions and issues addressed by art therapy, we extracted all scales and measures from the studies included in our review and assigned them to broader categories.

If a study had more than 1 method of measuring the same end point, we only included 1 measure for this end point, selected either based on popularity of the measure or interpretability of the score. Studies with an attrition of more than 50% were excluded from the analysis.

#### Search Strategy

To create a conclusive overview, we did not apply any language filters or date of publication limits. The search consisted of 3 elements, which were combined.^10^ The first element referred to the art, the second element referred to the therapeutic use of the art, and the third consisted of an adapted RCT filter.^13,14^ The search strategy was adapted according to the functionality of the individual databases, and the literature search syntax was peer reviewed by ^2^ of us (R.J. and A.B.) according to the Peer Review of Electronic Search Strategies guideline.^15^

Following the Cochrane recommendations, we searched the Cochrane library, MEDLINE, and Embase (through Ovid). In addition, we searched CINAHL, ERIC, American Psychological Association PsycArticles, American Psychological Association PsycInfo, PSYNDEX (all through EBSCOHost), the German Clinical Trials Register, and ClinicalTrials.gov. At the time of our search, the World Health Organization International Clinical Trials Registry Platform was not accessible. We also conducted a hand search of the *Journal of Creative Arts Therapies*. All systematic searches were conducted March 8 and 9, 2021.

### Study Selection

The study selection followed a 2-step process, aided by Covidence software^16^; first, all titles and abstracts were screened, and second, relevant studies were identified through thorough review of the full text. Both steps were done independently: the title and abstract screening (R.J., W.A.G., and A.B.) and the full text screening (R.J. and W.A.G.). All disagreements were resolved by a subsequent discussion.

The references of identified reviews were screened for studies that we might have missed. Any protocols for planned studies we found were matched to the published study for later bias evaluation and added to the list of ongoing studies.

### Data Extraction and Categorization

Information on the intervention (materials used, frequency, duration, and therapist training), control group, participants (age, sex, and indication), study characteristics (country of study and study arms), outcome (group size, scale description, and preintervention and postintervention data), data regarding possible bias, and other information were extracted by 1 of us (R.J. or C.K.) using a data extraction form (eAppendix in Supplement 1).

For better comparability, we tagged the outcomes across all studies with outcome tags, which we aggregated in a second step into 6 broader categories. A similar approach was used for the recorded treatment indications. Furthermore, we categorized the included study populations into 3 age groups according to the patients’ median, mean, or range of age, depending on how the data were reported. If participants spanned more than 1 age category, we labeled that study in accordance with the greatest numbers of its members. We defined children and adolescents as a study population younger than 18 years, adults as individuals aged between 18 and 65 years, and older adults as those older than 65 years.

If there were multiple control groups that could not be combined due to missing data (eg, combined SDs), we extracted the less-specific control group (eg, treatment as usual) to ensure greater compatibility across studies. If there were multiple follow-up measures of an outcome, we only considered the first measurement after the intervention had stopped to ensure comparability.

### Bias Evaluation

Bias evaluation was performed by 1 of us (R.J. or C.K.) using an adapted version of the Cochrane risk-of-bias tool for randomized trials.^17,18^ The evaluated domains of bias were the randomization process, deviations from intended interventions, missing outcome data, measurement tool used, measurement of the outcome, selection of the reported result, and other factors.

### Statistical Analysis

We performed random-effects meta-analyses, with standardized mean difference (SMD) as pooled-effect size and 2-sided 95% CIs. Outcomes with sufficient data are displayed in forest plots. To be displayed, each study outcome had to report at least the number of participants in the study and the mean (SD) of the outcome for the control and intervention groups separately, measured after the intervention. Likert scales with 5 or more levels, as well as scales with counts, were treated as continuous data for the analysis.

We summarized outcomes reported as either change from baseline or posttest separately, following standard practice. If both were reported, we used change from baseline. Change from baseline represents the change of variables after the AVAT treatment compared with a baseline level determined before the intervention, whereas posttest measures are observations after the intervention. Given the heterogeneity of the outcomes, we applied a random-effects model for the meta-analyses. We planned to perform a meta-regression including patient age, publication date, intervention duration, therapist qualification, treatment setting, type of AVAT, and country.

**Figure 2.**
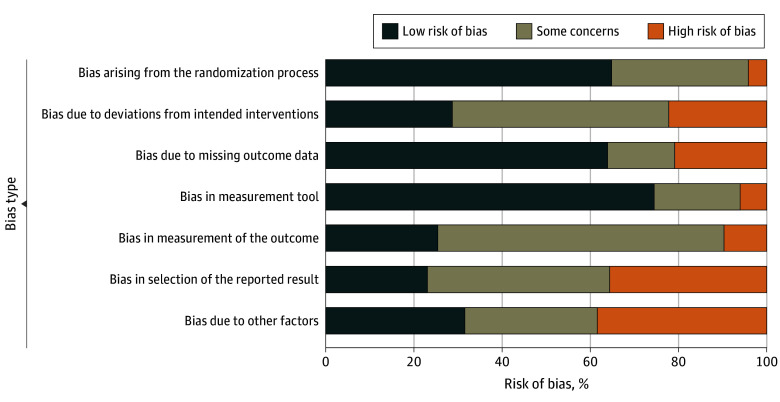
Bias as Assessed by the Adapted Cochrane Risk of Bias Tool for Randomized Trials for Outcomes of Studies Included in the Analysis

The analysis was conducted from April 24 to September 8, 2023, using RStudio, version 4.3.0 (R Foundation for Statistical Computing).^19^ Specifically, we used the package meta to calculate the meta-analysis and create the forest plots for all outcomes with sufficient and adequate outcome data.^19,20^ We chose Hedges *g* as the effect size and used the package’s default random-effects model DerSimonian-Laird. For the creation of the risk-of-bias plot (Figure 2), we used the packages robvis^21^ and ggplot2.^22^

## Results

### Results of Literature Search

Our search yielded 1368 results in the Cochrane library: 1223 through Ovid, 407 through EBSCOhost, 78 from Clinicaltrials.gov, and 10 from the German Clinical Trials Register. A further 18 studies were added after reference screening during the literature-screening stage. Of the resulting 3104 reports, 855 were duplicates and were removed using EndNote.^23^ The full study selection process can be seen in Figure 1.^11^ We translated all non-English and non-German records (including French, Chinese, Korean, and Turkish articles) during the screening process using Google Translate^24^ and DeepL Translator.^25^ There were 5 reports that could not be translated due to their format and resulting technical limitations (2 Persian, 2 Hebrew, and 1 Korean), and were thus excluded.

### Included Studies

We identified 75 reports of 69 studies that were eligible for this review^3,26,27,28,29,30,31,32,33,34,35,36,37,38,39,40,41,42,43,44,45,46,47,48,49,50,51,52,53,54,55,56,57,58,59,60,61,62,63,64,65,66,67,68,69,70,71,72,73,74,75,76,77,78,79,80,81,82,83,84,85,86,87,88,89,90,91,92,93,94,95,96,97,98^ (eTable 1 in Supplement 1). Some publications reported the results of 2 studies in 1 article (eg, Gussak et al^46^), whereas the results of other studies were reported in several publications (eg, Öster et al,^70,71,72^ Svensk et al,^75^ and Thyme et al^76^). When there were multiple reports of the same study, we included all outcome measures that were not duplicates. The article title and a brief description of the study population, intervention, and control group are presented in the eTable 1 in Supplement 1 , while a more-detailed description of the characteristics of the included studies, including age of participants, country of publication, scale of the outcome, total hours of intervention, level of bias, and characteristics of intervention and control groups, are displayed in the eTable in Supplement 2.

### Characteristics of Included Studies

#### Age of Study Population

Overall, most studies included an adult population (n = 31), followed by children and adolescents (n = 17) and older adults (n = 17); approximately 4200 participants were included. The age range was 4 to 96 years. Four studies examined the effect of AVAT on children together with their parents.

#### Countries

We included studies from 21 known countries; 1 study did not specify its location. Most studies were conducted in the US (23), followed by the UK (5), Iran (5), and Sweden (4). Japan, Israel, Germany, and China each contributed 3 trials. Furthermore, 2 studies were conducted in each of the following countries: the Netherlands, Turkey, Taiwan, South Korea, India, and France, and 1 study each was carried out in Iraq, Italy, Brazil, Russia, Tanzania, Australia, and Indonesia.

#### Comparators

The included control groups were very diverse and subsequently classified into 6 categories. These control categories were treatment as usual (25), other (17), attention control (10), no intervention (10), waiting list control (5), and not specified (2).

#### Included Interventions

The included studies used a wide variety of art therapy interventions, which we clustered into the 7 following categories according to the materials used: drawing/sketching (48), painting (43), arts and crafts (23), sculpting (17), not specified (12), coloring-in/mandala (7), and other (7). Most interventions fell into 2 or more of the categories. Most AVAT interventions (49) included a therapeutic element, 17 did not, and for the remaining 3 it was unclear.

#### Ongoing Studies

We identified 23 study descriptions of ongoing research projects that fulfilled our criteria of AVAT. An overview of these studies can be found in eTable 2 in Supplement 1.

### Treatment Indications for AVAT

Most studies investigated AVAT received by patients with mental health problems (37 studies), followed by patients with neurologic symptoms (13 studies), art therapy as prevention (10 studies), or for patients with other somatic challenges (9 studies).

### Outcomes Treated With AVAT

We identified 356 end point measures of 48 different outcome categories in the studies. Most outcome measures focused on depression, anxiety, self-esteem, social adjustment, and quality of life. Most frequently, AVAT was used to alleviate psychiatric symptoms (86 measures), improve psychological well-being (77), reduce social and behavioral problems (63), and improve cognitive function (16) or other somatic symptoms (eg, pain, asthma exacerbations, and hand function) (17). Seven measures (eg, treatment satisfaction or intervention costs) fit in none of those categories.

Of the identified 69 studies with 356 end point measures, we included 50 studies with 217 end point measures in the meta-analysis. Reasons for omitting end points included missing posttest means or SDs (96), a participant dropout rate of more than 50% (9), an ordinal (2) or dichotomous (2) outcome, and other reasons, such as measures of subscales when there was a total score of the instrument provided (30).

### Association of AVAT and Health Outcomes

A total of 217 outcomes of 50 studies from 53 publications, including data from 2766 patients were included in 2 meta-analyses^4,26,27,28,30,31,33,35,36,37,38,39,41,42,44,45,46,47,48,50,51,52,53,55,56,57,59,60,61,62,63,64,66,67,69,71,73,75,76,79,80,81,82,83,84,86,90,91,92,96,97,98,99^ (eTable 1 in Supplement 1). Of the total 217 outcomes, 18% favored AVAT, 1% favored the control group, and 81% showed no effect.

We created forest plots as a visual representation of the results (eFigure 1 and eFigure 2 in Supplement 1). These meta-analyses are explorative and thus nonconfirmatory. In the change from baseline random-effects meta-analysis, AVAT was associated with more positive health outcomes than the control groups (SMD, 0.38; 95% CI, 0.26-0.51). Results should be interpreted with caution due to substantial heterogeneity (*I*^2^ = 71%; τ^2^ = 0.23; *P* < .001).

The posttest random-effects meta-analysis also favored outcomes of disorders treated with AVAT over the control groups (SMD, 0.19; 95% CI, 0.12-0.26). Heterogeneity was substantial (*I*^2^ = 53%; τ^2^ = 0.08; *P* < .001).

### Subgroups

We conducted some explorative subgroup analyses. We divided the data by age groups and found, based on visual inspection of the forest plot, the outcomes of children improved slightly more on average than the other 2 age groups. When looking at the control groups, we could see that this seemed to be especially true when comparing AVAT with no intervention or treatment as usual (posttest) and waiting list (change from baseline) comparison groups, and that interventions with a therapeutic element might lead to a slightly more favorable total result.

### Meta-Regression

We calculated a meta-regression with the 9 moderators we predefined in the protocol. The meta-regression yielded inconclusive results due to the large number and variety of included factors.

### Risk of Bias Assessment

Bias in the measurement of the outcome, selective reporting bias, and other sources of bias were the most common causes for bias in the measurement of the outcomes. While many studies displayed low bias in multiple categories, all studies had at least 1 source of unclear or high bias. The eTable in Supplement 2 provides a detailed list of all assessed bias, including for missing outcome data. The funnel plots indicate a slight publishing bias (eFigure 3 and eFigure 4 in Supplement 1).

## Discussion

Overall, AVAT was associated with an improvement of health outcomes, especially in the area of mental health or when treating somatic conditions that may be associated with impaired mental health. This aligns with our practical observations made in clinical settings, where AVAT seems to be frequently associated with the management of mental health issues.

Multiple factors are of special relevance when discussing the results of this review. One of these factors involves the properties of the AVAT interventions. While it was exceeding the scope of this review to separate the effects of the different art types from each other to estimate outcomes, the presence or absence of a therapeutic element seems to be of particular relevance to the intervention. This observation aligns with the principles of patient-centered care, emphasizing the critical role of therapeutic engagement in the healing process.

A notable observation is the regional difference in AVAT research, with more studies conducted in the US and UK, possibly indicating differences in AVAT use. This may hint at the possible influence of structural and political factors shaping research priorities. With the benefits of AVAT, these findings underscore the need to promote equitable health care access by extending the benefits of AVAT to diverse populations, regardless of regional boundaries.

To further the quality of art therapy research and thereby lay the foundation for a future integration into standard care, it might be beneficial to establish guidelines that enhance research consistency and comparability. These guidelines should include standards for reporting AVAT and control interventions, thereby facilitating the future synthesis of treatment effects. Furthermore, the guidelines should incorporate a framework on suitable control interventions to avoid methodologic flaws. Such improvements would not only increase the research quality of the field but also provide a better foundation for evidence-based clinical decision-making.

### Limitations

This study has limitations. While this meta-analysis exclusively focused on RCTs, a triangulation of different methods is valuable when studying a complex and multifaceted intervention such as art therapy. Furthermore, the heterogeneity of the control groups posed unique challenges when examining AVAT. Several studies had to be excluded because the control group’s engagement included AVAT and therefore could not serve as a valid comparator. But even the remaining control interventions often contained therapy elements, such as psychotherapy, pharmaceuticals, and therapy dog encounters. Some complex treatment-as-usual interventions may have contained elements of AVAT. These potentially very effective control interventions might dilute the true effects of AVAT interventions in our data and have probably contributed to the observed heterogeneity. The observed association of AVAT and improved health outcomes in our data, despite these strong comparators, attests to the potential of AVAT.

## Conclusions

In this systematic review and meta-analysis, we found an overall benefit associated with AVAT interventions with the potential to improve various patient outcomes, particularly in the area of mental health. Those positive tendencies might even increase in magnitude with improving study quality. To reach that goal, international collaboration and harmonization of research methods are important.

## Notes

Supplement 1. **eAppendix.** Data Extraction Form **eTable 1.** Characteristics of the Included Studies, Including the Short ID, Title, a Brief Description of the Study Population, Intervention and Control Group **eTable 2.** Ongoing or Recently Published Studies That Investigate the Effectiveness of AVAT **eFigure 1.** Forest Plot of the Effects of AVAT for Studies Presenting Results as Change From Baseline **eFigure 2.** Forest Plot of the Effects of AVAT for Studies Presenting Results as Post-test **eFigure 3.** Funnel Plot for Change From Baseline Outcomes
eReferences

Supplement 2. **eTable.** Detailed Description of Study Characteristics

Supplement 3.
Data Sharing Statement
