# Supplementary material for: Active Visual Art Therapy and Health Outcomes: A Systematic Review and Meta-Analysis
Source: JAMA Netw Open. 2024 Sep 12;7(9):e2428709. doi: 10.1001/jamanetworkopen.2024.28709 (PMC11393726; doi:10.1001/jamanetworkopen.2024.28709)
Supplement: Supplement 1. — eAppendix. Data Extraction Form eTable 1. Characteristics of the Included Studies, Including the Short ID, Title, a Brief Description of the Study Population, Intervention and Control Group eTable 2. Ongoing or Recently Published Studies That Investigate the Effectiveness of AVAT eFigure 1. Forest Plot of the Effects of AVAT for Studies Presenting Results as Change From Baseline eFigure 2. Forest Plot of the Effects of AVAT for Studies Presenting Results as Post-test eFigure 3. Funnel Plot for Change From Baseline Outcomes eReferences [file jamanetwopen-e2428709-s001.pdf]

Supplementary Online Content

Joschko R, Klatte C, Grabowska WA, Roll S, Berghöfer A, Willich SN. Active visual art therapy and health outcomes: a meta-analysis. *JAMA Netw Open*. 2024;7(8):e2428709. doi:10.1001/jamanetworkopen.2024.28709

eAppendix. Data Extraction Form

eTable 1. Characteristics of the Included Studies, Including the Short ID, Title, a Brief Description of the Study Population, Intervention and Control Group

eTable 2. Ongoing or Recently Published Studies That Investigate the Effectiveness of AVAT

eFigure 1. Forest Plot of the Effects of AVAT for Studies Presenting Results as Change From Baseline

eFigure 2. Forest Plot of the Effects of AVAT for Studies Presenting Results as Post-test

eFigure 3. Funnel Plot for Change From Baseline Outcomes

eReferences

This supplementary material has been provided by the authors to give readers additional information about their work.

© 2024 Joschko R et al. *JAMA Network Open.*

eAppendix: Data extraction form.

For each included study we extracted the following:

| Study | Year | same study as | title | indication tag | population tag | population status | outcome tag | other | country | setting | age | age range | total women | total men | study design | study arms | outcome category | construct | scale | scale description | scaling | continuous vs dichotomous | imputation | ITT vs PP | N int randomised | N control randomised | pre intervention mean | pre intervention SD | pre intervention N | pre control mean | pre control SD | pre control N | total N beginning | post intervention mean | post intervention SD | post intervention N | post control mean | post control SD | post control N | weeks since baseline | MD intervention | SD MD intervention | MD control | SD MD control | total N end of study | Cohen's d reported | tendency | other measures | control group | art | materials used | material abbreviation | group type | therapeutical element | therapist qualification | mins per session | sessions per week | weeks of intervention | total minutes of intervention | timing | aim | funding | randomisation | bias from randomisation | randomisation in text | deviation from intended intervention bias | deviation from intended intervention bias in text | dropout missing outcome data bias | dropout missing outcome bias in text | dropout intervention group in percent | dropout control in percent | total dropout in percent | bias in measurement tool | bias in measurement tool explanation | bias in measurement of the outcome | bias in measurement outcome in text | selective reporting bias | selective reporting explanation | other bias | other bias description |

© 2024 Joschko R et al. *JAMA Network Open.*

eTable 1: Characteristics of the included studies, including the short ID, title, a brief description of the study population, intervention and control group.

Papers reporting the same study share a bold outline. Studies included in the review but not the meta-analysis have a grey background.

Study ID	Title	Population	Materials	Therapeutical element	Control group tag
Abbing 2019 a[1]	The Effectiveness of Art Therapy for Anxiety in Adult Women: A Randomized Controlled Trial.	Adult women with moderate to severe anxiety symptoms	Sculpting, Painting, Drawing/sketching	yes	Waitlist
Abbing 2019 b[2]	Anxiety reduction through art therapy in women. Exploring stress regulation and executive functioning as underlying neurocognitive mechanisms.	See above	Sculpting, Painting, Drawing/sketching	yes	Waitlist
Abdulah 2018 [3]	Effectiveness of group art therapy on quality of life in paediatric patients with cancer: a randomized controlled trial	Children undergoing chemotherapy	Painting, Arts and crafts, Drawing/sketching	no	TAU
Barfarazi 2018 [4]	Evaluating the Effect of Painting Therapy on Happiness in the Elderly	Older adults in elderly care centers	Painting	yes	TAU
Bazargan 2016 [5]	The Effectiveness of Art Therapy in Reducing Internalizing and Externalizing Problems of Female Adolescents	Teenage girls with internalizing (n=30) and externalizing (n=30) problems	Painting, Drawing/sketching	yes	Not specified
Beebe 2010 [6]	A randomized trial to test the effectiveness of art therapy for children with asthma	School children with asthma	Painting, Drawing/sketching, Sculpting, Arts and crafts	yes	Waitlist
Beh-Pajooh 2018 [7]	The effectiveness of painting therapy program for the treatment of externalizing behaviours in children with intellectual disability	Male children with intellectual disability in special need schools	Painting, Drawing/sketching	no	No intervention

© 2024 Joschko R et al. *JAMA Network Open.*

Study ID	Title	Population	Materials	Therapeutical element	Control group tag
Blomdahl 2018 [8]	A manual-based phenomenological art therapy for individuals diagnosed with moderate to severe depression (PATd): a randomized controlled study	Adults with moderate to severe depression without psychotic symptoms	Painting, Drawing/sketching	yes	TAU
Borchers 1985 [9]	Do gains made in group art therapy persist? A study with aftercare patients	Adults with severe chronic mental illness after in-patient treatment	Not specified	yes	TAU
Bozzatello 2019 [10]	Effectiveness of psychosocial treatments on symptoms and functional domains in schizophrenia spectrum disorders: a prospective study in a real-world setting.	Adults with schizophrenia and related symptoms	Not specified	yes	Other: befriending group.
Broome 2001 [11] adolescents	An intervention to increase coping and reduce health care utilization for school-age children and adolescents with sickle cell disease	Schoolaged children/ adolescents who attended an information program about living with sickle cell disease	Drawing/sketching, Painting, Not specified	yes	TAU and CBT for pain
Broome 2001 [11] children	An intervention to increase coping and reduce health care utilization for school-age children and adolescents with sickle cell disease	Schoolaged children/ adolescents who attended an information program about living with sickle cell disease	Drawing/sketching, Painting, Not specified	yes	TAU and CBT for pain
Cetinkaya 2019 [12]	The Effect of Ceramic Painting on the Life Satisfaction and Cognitive Status of Older Adults Residing in a Nursing Home	Older adults (65+), who were residing in nursing home, without dementia	Colouring in/mandala, Painting	no	No intervention

© 2024 Joschko R et al. *JAMA Network Open.*

Study ID	Title	Population	Materials	Therapeutical element	Control group tag
Ching-Teng 2019 [13]	Positive effects of art therapy on depression and self-esteem of older adults in nursing homes	Older adults in long-term care institutes with intact mental functions and mild depression	Painting, Drawing/sketching, Arts and crafts, Other	yes	TAU
Choi 2020 [14]	The effects of combining art psychotherapy with pharmacotherapy in treating major depressive disorder: randomized control study	Adults with pharmacotherapy for major depressive disorder (MDD)	Drawing/sketching	yes	TAU
Ciasca 2018 [15]	Art therapy as an adjuvant treatment for depression in elderly women: a randomized controlled trial	Elderly women (60+) with major depressive disorder	Painting, Drawing/sketching, Arts and crafts, Sculpting	yes	TAU
Crawford 2012 [16]	Group art therapy as an adjunctive treatment for people with schizophrenia: multicentre pragmatic randomised trial	Adults with schizophrenia	Not specified	yes	TAU
Leurent 2014 [17]	Moderating factors for the effectiveness of group art therapy for schizophrenia: secondary analysis of data from the MATISSE randomised controlled trial	Adults with schizophrenia	Not specified	yes	TAU
Decker 2018 [18]	Quantitatively Improved Treatment Outcomes for Combat-Associated PTSD With Adjunctive Art Therapy: randomized Controlled Trial	U.S. veterans with PTSD	Drawing/sketching	yes	Other: Cognitive processing therapy and supportive psychotherapy plus TAU (pharmacological therapy for PTSD and depression)

© 2024 Joschko R et al. *JAMA Network Open.*

Study ID	Title	Population	Materials	Therapeutical element	Control group tag
Ellis-Hill 2019 [19]	HeART of Stroke: randomised controlled, parallel-arm, feasibility study of a community-based arts and health intervention plus usual care compared with usual care to increase psychological well-being in people following a stroke	Max. 2 years poststroke, community-dwelling adults and older adults	Painting, Drawing/sketching, Sculpting, Arts and crafts	yes	TAU
Gussak 2006 [20]	Effects of art therapy with prison inmates: a follow-up study	Adult male prisoners, most taking medication for mental illness	Drawing/sketching, Sculpting	yes	No intervention = TAU
Gussak 2009 men [21]	The effects of art therapy on male and female inmates: Advancing the research base	Adult male prisoners, a majority having an axis 1 diagnosis and taking medication	Arts and crafts, Drawing/sketching	yes	No intervention = TAU
Gussak 2009 women [21]	The effects of art therapy on male and female inmates: Advancing the research base	Adult female prisoners, a majority having an axis 1 diagnosis and taking medication	Arts and crafts, Drawing/sketching	yes	No intervention = TAU
Haeyen 2018 [22]	Efficacy of Art Therapy in Individuals With Personality Disorders Cluster B/C: a Randomized Controlled Trial	Adults with a primary diagnosis of at least one Axis II Personality Disorder cluster B and/or C or a personality disorder, not otherwise specified	Not specified	yes	Waitlist

© 2024 Joschko R et al. *JAMA Network Open.*

Study ID	Title	Population	Materials	Therapeutical element	Control group tag
Hattori 2011 [23]	Controlled study on the cognitive and psychological effect of coloring and drawing in mild Alzheimer's disease patients	Older adults (65–85 years) who consulted the outpatient clinic and showing mild impairment of cognitive function (Mini-Mental State Examination score of 20 or higher)	Colouring in/mandala, Drawing/sketching	maybe	Other: "learning therapy" involving simple calculation
Hill 2016 [24]	Improving the Short-Term Affect of Grieving Children Through Art	Children (6-13years) who had experienced the death of a family member (either parent or sibling)	Painting	no	Other: Attention control consisting of puzzling
Hsiao 2020 [25]	Effects of Art and Reminiscence Therapy on Agitated Behaviors Among Older Adults With Dementia	Older adults (55+) with mild/ moderate/ severe dementia (MMSE 18 +)	Painting, Drawing/sketching, Sculpting, Arts and crafts	probably not	TAU
Ilali 2018 [26]	Impact of Art-Based Life Review on Depression Symptoms Among Older Adults	Older adults (60+) with depression (Short Geriatric Depression Scale 11 or lower), not taking antidepressants, or stable on antidepressants for min. 3 months, and with an abbreviated Mental Test of 7 or higher	Painting, Drawing/sketching	yes	TAU

© 2024 Joschko R et al. *JAMA Network Open.*

Study ID	Title	Population	Materials	Therapeutical element	Control group tag
Jouybari 2018 [27]	Comparison of the effect of narrative writing and art therapy on maternal stress in neonatal intensive care settings	Mothers with a premature infant (born before 38 weeks' gestation) in NICU (neonatal intensive care unit)	Drawing/sketching	no	TAU
Katz 2014 [28]	The effect of drawing on children's experiences of investigations following alleged child abuse	Children who were alleged victims of sexual abuse (by a non-family member)	Drawing/sketching	yes	No intervention
Khedekar 2017 [29]	Use of art as therapeutic intervention for enhancement of hand function in patients with rheumatoid arthritis: a pilot study	Adults with rheumatoid arthritis Class II / III	Painting, Sculpting, Arts and crafts	no	TAU
Kim 2013 [30]	A randomized, controlled study of the effects of art therapy on older Korean-Americans' healthy aging	Older Korean-American adults with MMSE > 25	Painting, Drawing/sketching, Sculpting	yes	TAU
Kline 2020 [31]	Randomized Trial of Therapy Dogs Versus Deliberative Coloring (Art Therapy) to Reduce Stress in Emergency Medicine Providers	Emergency Medicine providers working shift work	Colouring in/mandala	no	Other: Therapy dog
Kopytin 2013 [32]	Humor, Self-Attitude, Emotions, and Cognitions in Group Art Therapy With War Veterans	Hospitalised war veterans over 55 years with nonpsychotic mental disorders	Painting, Drawing/sketching	yes	TAU
Lalingkar 2020 [33]	Effect of recreational therapy on attention and depression in school children with learning difficulties	School children with any learning difficulty, MARS attention score > 50 and depression score > 13	Colouring in/mandala, Sculpting	no	TAU

© 2024 Joschko R et al. *JAMA Network Open.*

Study ID	Title	Population	Materials	Therapeutical element	Control group tag
Laurer 2015 [34]	Effect of Art Production on Negative Mood and Anxiety for Adults in Treatment for Substance Abuse	Adults with substance abuse or dependence disorder	Painting, Drawing/sketching	no	Other: Viewing and sorting art
Lawson 2016 [35]	Effects of Making Art and Listening to Music on Symptoms Related to Blood and Marrow Transplantation	Adults receiving blood and marrow transplantations	Painting, Drawing/sketching	no	TAU
Liu 2018 [36]	Examining the effectiveness of solution-focused art therapy (SF-AT) for sleep problems of children with traumatic experience	Children with traumatic experience	Arts and crafts, Other	yes	TAU
Lock 2017 [37]	Feasibility Study Combining Art Therapy or Cognitive Remediation Therapy with Family-based Treatment for Adolescent Anorexia Nervosa	Adolescents with anorexia nervosa who have obsessive–compulsive (OC) features and respond poorly to family-based treatment (FBT).	Drawing/sketching, Not specified	yes	Other: CBT and family based treatment
Masika 2021 [38]	Can Visual Art Therapy Be Implemented With Illiterate Older Adults With Mild Cognitive Impairment? A Pilot Mixed-Method Randomized Controlled Trial	Older adults with mild cognitive impairment (MCI) with no literacy and increased risk of progression to dementia	Drawing/sketching	yes	Other: Attention control; health education
McCaffrey 2007 [39]	The effect of healing gardens and art therapy on older adults with mild to moderate depression.	Older adults with depression (self-diagnosed or diagnosed by a healthcare provider), who are able to walk 7-10 miles	Drawing/sketching	yes	Other: Attention control; garden walks

© 2024 Joschko R et al. *JAMA Network Open.*

Study ID	Title	Population	Materials	Therapeutical element	Control group tag
Montag 2014 [40]	A pilot RCT of psychodynamic group art therapy for patients in acute psychotic episodes: feasibility, impact on symptoms and mentalising capacity	Adults in inpatient treatment with schizophrenia	Painting, Drawing/sketching, Sculpting	yes	TAU
Morris 2019 [41]	Art participation for psychosocial wellbeing during stroke rehabilitation: a feasibility randomised controlled trial	Medically stable stroke survivors participating in usual rehabilitation therapies with a planned rehabilitation duration of at least three weeks	Painting, Drawing/sketching, Sculpting, Arts and crafts	yes	TAU
Narme 2012 [42]	Nonpharmacological treatment for Alzheimer's disease: comparison between musical and non-musical interventions	Adults with moderate to severe Alzheimer's disease	Painting	yes	Other: music therapy
Omizo 1989 [43]	Art Activities to Improve Self-Esteem Among Native Hawaiian Children	Hawaiian or part-hawaiian children from lower-middle to lower socioeconomic backgrounds	Painting, Drawing/sketching, Sculpting, Arts and crafts, Other	yes	No intervention

© 2024 Joschko R et al. *JAMA Network Open.*

Study ID	Title	Population	Materials	Therapeutical element	Control group tag
Öster 2006 [44]	Art therapy improves coping resources: A randomized, controlled study among women with breast cancer	Women with newly diagnosed, nonmetastasic breast cancer, who don't have dementa or severe psychiatric illness, receiving postoperative radiotherapy. 9 women of the study group and 10 women of the control group also received chemotherapy.	Painting, Drawing/sketching, Arts and crafts	yes	TAU
Öster 2007 [45]	Art therapy for women with breast cancer: The therapeutic consequences of boundary strengthening	See above	Painting, Drawing/sketching, Arts and crafts	yes	TAU
Öster 2014 [46]	Art therapy during radiotherapy - A five-year follow-up study with women diagnosed with breast cancer	See above	Painting, Drawing/sketching, Arts and crafts	yes	TAU
Thyme 2009 [47]	Individual brief art therapy can be helpful for women with breast cancer: A randomized controlled clinical study	See above	Painting, Drawing/sketching, Arts and crafts	yes	TAU
Svensk 2009 [48]	Art therapy improves experienced quality of life among women undergoing treatment for breast cancer: A randomized controlled study	See above	Painting, Drawing/sketching, Arts and crafts	yes	TAU

© 2024 Joschko R et al. *JAMA Network Open.*

Study ID	Title	Population	Materials	Therapeutical element	Control group tag
Pongan 2017 [49]	Can Musical or Painting Interventions Improve Chronic Pain, Mood, Quality of Life, and Cognition in Patients with Mild Alzheimer's Disease? Evidence from a Randomized Controlled Trial	Older adults (60+) with probable Alzheimer disease at minor cognitive disorder or mild major cognitive disorder stage	Painting	probably not	Other: choral singing
Qin 2020 [50]	Effect of music therapy intervention on physical functions and mental health of patients with ankylosing spondylitis	Hospitalized patients with ankylosing spondylitis (AS)	Painting, Drawing/sketching	probably not	TAU
Agbaria 2020 [51]	Acquiring social and cognitive skills in an intervention for Arab parents of children with intellectual developmental disability accompanied by behavioral conditions.	Parents of children with minor IDD (intellectual developmental disability) who attend a special education school	Painting	maybe	Other: CBT
Rajendran 2020 [52]	Randomized Controlled Trial of Adult Therapeutic Coloring for the Management of Significant Anxiety in the Emergency Department	Adult patients waiting in the emergency department	Colouring in/mandala	no	Other: Attention control; Pen and Paper
Ramirez 2020 [53]	Investigating impact: the effects of school-based art therapy on adolescent boys living in poverty	Male adolescents living in poverty and attending a private school	Drawing/sketching, Sculpting, Arts and crafts		Other: Attention control; doing homework assigned by other teachers or interacting with peers
Rao 2009 [54]	Art therapy for relief of symptoms associated with HIV/AIDS	People living with HIV/AIDS	Painting, Drawing/sketching, Arts and crafts, Other	yes	Other: Attention control; videotape on the uses of art therapy

© 2024 Joschko R et al. *JAMA Network Open.*

Study ID	Title	Population	Materials	Therapeutical element	Control group tag
Regev 2005 [55]	The psychological benefits of artwork: the case of children with learning disorders	Primary-school children with learning disorders	Painting, Sculpting, Arts and crafts	yes	No intervention
Richardson 2007 [56]	Exploratory RCT of art therapy as an adjunctive treatment in schizophrenia	Outpatient adults (mental health service users) with a diagnosis of chronic schizophrenia, not in other psychological treatment.	Not specified	yes	TAU
Robinson 2018 [57]	Randomized Trial Evaluating the Effectiveness of Coloring on Decreasing Anxiety Among Parents in a Pediatric Surgical Waiting Area	Parents of children undergoing surgery	Colouring in/mandala	no	No intervention
Rosal 1993 [58]	Comparative group art therapy research to evaluate changes in locus of control in behavior disordered children	4th, 5th and 6th graders of a primary school in a housing commission, with moderate to severe behaviour problems	Painting, Drawing/sketching, Sculpting, Arts and crafts	yes	Not specified, probably no intervention
Roswiyani 2020 [59]	Art activities and qigong exercise for the well-being of older adults in nursing homes in Indonesia: a randomized controlled trial	Older adults living in nursing homes for at least 3 month, with a Mini-Mental State Examination (MMSE) score 18 or more	Drawing/sketching, Colouring in/mandala, Arts and crafts, Painting	yes	No intervention
Rusted 2006 [60]	A multi-centre randomized control group trial on the use of art therapy for older people with dementia	Older adults with dementia	Not specified	yes	Other: Attention control; activity groups not involving art or occupational therapy elements

© 2024 Joschko R et al. *JAMA Network Open.*

Study ID	Title	Population	Materials	Therapeutical element	Control group tag
Schrade 2011 [61]	Physiological effects of mandala making in adults with intellectual disability	Adults and older adults with mental disability who attended a day program at a non-profit agency	Drawing/sketching	no	Other: Attention control consisting of puzzling and table games
Schreier 2005 [62]	Posttraumatic stress symptoms in children after mild to moderate pediatric trauma: a longitudinal examination of symptom prevalence, correlates, and parent-child symptom reporting	Children and adolescents (7-17) who were hospitalized for at least 24h after a physical injury, and showing at least mild trauma symptoms	Drawing/sketching	yes	TAU
Siegel 2016 [63]	Expressive arts therapy for hospitalized children: a pilot study measuring cortisol levels	Children and adolescents (3-17) who are hospitalised for various reasons (dog attack, cancer, acute asthma, perforated appendicitis, encopresis etc.)	Other	yes	Waitlist
Yount 2013 [64]	Expressive arts therapy for hospitalized children: a pilot study measuring cortisol levels	See above	Other	yes	Waitlist
Sommer 2001 [65]	Creative-dynamic image synthesis: a useful addition to the treatment options for impotence	Adult men with erectile dysfunction of no known organic cause	Drawing/sketching	yes	Other
Sterz 2013 [66]	Creative arts therapy improves quality of life in MS - Results of a randomized controlled trial during inpatient rehabilitation	Patients with multiple sclerosis during an inpatient rehabilitation	Painting, Sculpting	yes	Attention contrl

© 2024 Joschko R et al. *JAMA Network Open.*

Study ID	Title	Population	Materials	Therapeutical element	Control group tag
Sükrü 2018 [67]	The impact of a six-month interpersonal group psychotherapy on functionality of patients with schizophrenia in a community mental health center	Adults with early (onset within the last 5 years) schizophrenia receiving antipsychotic medication	Painting	maybe	Waitlist
Thyme 2007 [68]	The outcome of short-term psychodynamic art therapy compared to short-term psychodynamic verbal therapy for depressed women	Adult women with depression or dysthymic disorder, who are not using psychopharmacological treatment	Not specified, Drawing/sketching	yes	Other
Tibbetts 1990 [69]	Short-term art therapy with seriously emotionally disturbed adolescents	"Seriously emotionally disturbed" adolescents enrolled in a "Los Angeles County Office of Education Special Class Alternative"	Not specified	yes	Attention control
Wahlbeck 2020 [70]	Art Therapy and Counseling for Fear of Childbirth: a Randomized Controlled Trial	Pregnant adult women with a pronounced fear of childbirth	Painting	yes	TAU
Wu 2020 [71]	After the intervention after the intervention an evaluation and analysis of visual art therapy in the treatment of PTSD	Adults diagnosed with PTSD	Painting, Other	yes	TAU
Yamada 2010 [72]	A randomised clinical trial of a wellness programme for healthy older people	Community dwelling older adults taking part in a wellness programme	Arts and crafts	no	Other

© 2024 Joschko R et al. *JAMA Network Open.*

Study ID	Title	Population	Materials	Therapeutical element	Control group tag
Yan 2019 [73]	School bullying among left-behind children: the efficacy of art therapy on reducing bullying victimization	Left-behind children: children in rural areas whose parents left for more than 6 months to work in the city	Painting, Drawing/ sketching	yes	No intervention
Yu 2016 [74]	House-Tree-Person drawing therapy as an intervention for prisoners' prerelease anxiety	Male adult prisoners who will be released from prison in 2-6 months and potentially suffer from anxiety	Drawing/ sketching	yes	TAU

© 2024 Joschko R et al. *JAMA Network Open.*

eTable 2: Ongoing or recently published studies that investigate the effectiveness of AVAT

Reference	Title
Bacha 2019 [75]	Impact of art therapy on brain connectivity in recent post-stroke aphasia (ART-CONNECT)
Beauchet 2020 [76]	Effects of "Thursdays at the Museum" at the Montreal Museum of Fine Arts on the mental and physical health of older community dwellers: The art-health randomized clinical trial protocol
Chan 2021 [77]	A randomised controlled trial of expressive arts-based intervention for young stroke survivors
DRKS00012417 2020[78]	Art therapy as a preventive or interventional treatment for geriatric patients
DRKS00013301 2018 [79]	Art therapy to regulate hypertension - a quantitative, controlled study
DRKS00021092 2020 [80]	Older Adults in Nuremberg and art therapy
DRKS00022989 2020 [81]	Psycho-biological treatment effects of trauma-focused art therapy in patients with psychological and physical trauma consequences
Guillaume 2021	The effects of an 8-week knitting program on osteoarthritis symptoms in elderly women: A pilot randomized controlled trial
Guitard 2018	The knitting community-based trial for older women with osteoarthritis of the hands: design and rationale of a randomized controlled trial
IRCT20200104046002N1 2020 [82]	The effect of art therapy on anxiety and quality of life following pregnancy loss
ISRCTN96947690 2020 [82]	The effects of art therapy with clay on emotion regulation, stress, and hormone levels in secondary school students
Joly 2016 [83]	Impact of art therapy (AT) on fatigue and quality of life (QoL) during adjuvant external beam irradiation (EBI) in breast cancer patients (pts): a randomized trial
Lee 2019 [84]	T103. The effects of group arts therapy based on emotion management training on the emotional expression, alexithymia, depression and quality of life in patients with schizophrenia...2019 congress of the schizophrenia international research society, 10-14
Matus 2020 [85]	Impact of waiting room art intervention on pediatric patients' anxiety and cooperation with physician
Naveed 2020 [86]	A comparison of group art therapy in decreasing the depression level of leukaemia paediatric patients
NCT03820843 2019 [75]	Impact of Art Therapy on Brain Connectivity in Recent Post-Stroke Aphasia
NCT04195217 2019 [87]	Application of Art Therapy in Oncology: evaluation of the Symptomatic of Patients Suffering From a Cancerous Disease
NCT04493034 2020 [88]	Assessing the Benefit of Art & Music Therapy on Quality of Life in Patients With Breast Cancer
NCT04524260 2020 [89]	The Effects of Perioperative Painting Art Therapy (LOMÂ® Solution Centered Art Therapy) in Surgical Patients
NCT04548115 2020 [90]	ARTISAN 2.0: deconstructing the Integrative Efficacy of a Multimodal Art-based Intervention
NCT04714255 2022 [91]	Effectiveness of Distraction (Art Therapy) on Reducing Pain and Anxiety During Intravenous Cannulation
Pieper 2020 [92]	The UPGRADE study protocol: creative strengthening groups as an innovative preventive intervention for healthcare workers
Gohier 2019 [93]	Impact of Art Therapy on Alexithymia in People With Alcohol Use Disorder

eFigure 1. Forest plot of the effects of AVAT for studies presenting results as change from baseline

Outcome	AVAT Total	AVAT Mean	AVAT SD	Control Total	Control Mean	Control SD	SMD [95%−CI]
Anxiety[56] (Kim 2013)	25	13.17	5.47	25	−3.08	5.05	3.04 [2.21; 3.87]
Self−esteem[56] (Kim 2013)	25	4.24	2.42	25	−0.48	1.85	2.16 [1.45; 2.86]
Anxiety[31] (Beebe 2010)	11	15.60	10.83	11	−5.30	9.79	1.95 [0.90; 2.99]
Stress[98] (Yount 2013)	11	0.95	1.19	12	−0.50	0.67	1.47 [0.53; 2.41]
Self concept[31] (Beebe 2010)	11	12.09	5.66	11	−3.54	13.56	1.45 [0.49; 2.41]
QoL[26] (Abbing 2019 a)	24	8.00	5.28	23	1.48	3.92	1.37 [0.73; 2.02]
Depression[51] (Ilali 2018)	27	2.15	1.97	27	−0.00	1.47	1.22 [0.63; 1.80]
Eating disorder/Weight[63] (Lock 2017)	11	0.08	0.05	12	0.03	0.03	1.18 [0.28; 2.08]
Anxiety[78] (Rajendran 2020)	26	3.70	3.30	27	0.30	2.30	1.18 [0.59; 1.77]
QoL[31] (Beebe 2010)	11	9.73	13.71	11	−13.36	23.48	1.16 [0.24; 2.07]
Depression[64] (Masika 2021)	15	2.89	2.68	18	−0.00	2.71	1.04 [0.31; 1.78]
Anger[31] (Beebe 2010)	11	10.09	14.04	11	−2.30	9.66	0.99 [0.09; 1.88]
Anxiety[26] (Abbing 2019 a)	24	19.71	24.07	23	0.13	13.99	0.97 [0.37; 1.58]
Depression[45] (Gussak 2006)	16	7.81	9.81	13	−1.00	7.59	0.96 [0.18; 1.74]
Depression[41] (Ciasca 2018)	31	3.20	3.40	25	0.60	2.32	0.86 [0.31; 1.42]
Cognitive Performance[63] (Lock 2017)	10	0.34	0.74	11	−0.21	0.54	0.82 [−0.08; 1.72]
Hand mobility[55] (Khedekar 2017)	5	1.57	1.22	5	0.72	0.54	0.81 [−0.51; 2.13]
Self−efficacy/ Locus of Control[46] (Gussak 2009 women)	71	3.21	5.58	20	−0.70	4.37	0.73 [0.22; 1.23]
Obsessive−Cumpulsive Symptoms[63] (Lock 2017)	11	8.72	7.29	12	3.66	6.22	0.72 [−0.13; 1.57]
Depression[31] (Beebe 2010)	11	10.91	12.90	11	−1.30	19.71	0.71 [−0.16; 1.57]
Depression[41] (Ciasca 2018)	31	8.60	12.80	25	1.60	4.86	0.69 [0.14; 1.23]
PTSD/Trauma[62] (Liu 2018)	20	6.88	10.57	20	−1.12	12.69	0.67 [0.03; 1.31]
Depression[46] (Gussak 2009 men)	35	6.69	10.38	25	−0.12	9.80	0.66 [0.13; 1.19]
Depression[46] (Gussak 2009 women)	76	10.67	11.10	20	4.30	5.22	0.62 [0.12; 1.12]
Self−efficacy/ Locus of Control[67] (Morris 2017)	33	2.60	7.10	38	−1.50	6.60	0.59 [0.12; 1.07]
Self−efficacy/ Locus of Control[46] (Gussak 2009 men)	37	1.22	3.61	25	−1.04	4.04	0.59 [0.07; 1.11]
Depression[33] (Blomdahl 2018)	43	6.16	8.41	35	1.37	8.06	0.57 [0.12; 1.03]
Self regulation[26] (Abbing 2019 a)	24	13.04	19.46	23	3.70	12.13	0.56 [−0.02; 1.15]
Depression[85] (Roswiyani 2020)	63	3.71	6.68	65	−0.45	7.99	0.56 [0.21; 0.91]
Positive affect[67] (Morris 2017)	33	0.90	3.50	38	−1.50	4.90	0.55 [0.08; 1.03]
Depression[63] (Lock 2017)	9	10.66	11.09	10	4.50	12.96	0.49 [−0.43; 1.40]
Eating disorder/Weight[63] (Lock 2017)	11	2.10	1.38	12	1.51	0.95	0.48 [−0.35; 1.32]
Eating disorder/Weight[63] (Lock 2017)	11	9.82	6.37	12	6.17	8.75	0.46 [−0.37; 1.29]
Anxiety[41] (Ciasca 2018)	31	8.90	14.50	25	2.90	11.36	0.45 [−0.09; 0.98]
QoL[85] (Roswiyani 2020)	63	7.68	16.18	65	0.89	14.30	0.44 [0.09; 0.79]
Somatic Symptoms[67] (Morris 2017)	33	10.10	24.90	38	1.40	17.20	0.41 [−0.06; 0.88]
Eating disorder/Weight[63] (Lock 2017)	11	8.77	6.22	12	6.39	5.10	0.41 [−0.42; 1.23]
Pain[55] (Khedekar 2017)	5	−84.00	39.74	5	−102.00	42.07	0.40 [−0.86; 1.66]
Cognitive Performance[41] (Ciasca 2018)	31	0.60	1.70	25	0.10	0.80	0.36 [−0.17; 0.89]
Life Satisfaction[85] (Roswiyani 2020)	63	1.51	5.68	65	−0.28	4.34	0.35 [0.00; 0.70]
Hand mobility[55] (Khedekar 2017)	5	1.25	0.60	5	1.02	0.60	0.35 [−0.91; 1.60]
Self−efficacy/ Locus of Control[84] (Rosal 1993)	12	2.17	3.97	12	1.08	2.02	0.33 [−0.47; 1.14]
Self−esteem[67] (Morris 2017)	33	0.40	6.70	38	−2.10	8.40	0.32 [−0.15; 0.79]
Well−being[85] (Roswiyani 2020)	63	3.97	18.73	65	−2.08	18.56	0.32 [−0.03; 0.67]
QoL[85] (Roswiyani 2020)	63	5.55	15.24	65	1.30	11.43	0.31 [−0.03; 0.66]
Hand mobility[55] (Khedekar 2017)	5	0.87	0.08	5	0.78	0.36	0.31 [−0.94; 1.56]
Hand mobility[55] (Khedekar 2017)	5	−24.00	8.94	5	−28.00	14.83	0.29 [−0.96; 1.55]
Well−being[85] (Roswiyani 2020)	63	3.30	22.17	65	−1.35	15.10	0.24 [−0.10; 0.59]
Somatic Symptoms[85] (Roswiyani 2020)	63	3.97	22.58	65	−0.54	17.00	0.22 [−0.12; 0.57]
Anxiety[63] (Lock 2017)	9	3.62	9.30	10	1.30	11.51	0.21 [−0.69; 1.11]
Pain[85] (Roswiyani 2020)	63	5.97	30.67	65	1.14	16.00	0.20 [−0.15; 0.54]
Cognitive Performance[64] (Masika 2021)	15	3.47	2.73	18	3.00	3.16	0.15 [−0.53; 0.84]
QoL[55] (Khedekar 2017)	5	−0.66	0.46	5	−0.70	0.28	0.09 [−1.15; 1.34]
Psychiatric Symptoms[92] (Sukru 2018)	19	4.60	6.83	17	3.88	8.27	0.09 [−0.56; 0.75]
Psychiatric Symptoms[35] (Bozzatello 2019)	25	1.00	7.75	29	0.30	9.01	0.08 [−0.45; 0.62]
Social Adjustment[85] (Roswiyani 2020)	63	0.17	29.12	65	−0.77	19.39	0.04 [−0.31; 0.38]
Self−esteem[33] (Blomdahl 2018)	42	3.29	2.99	35	3.26	2.68	0.01 [−0.44; 0.46]
Hand mobility[55] (Khedekar 2017)	5	−190.00	62.05	5	−190.00	69.64	0.00 [−1.24; 1.24]
Self−efficacy/ Locus of Control[67] (Morris 2017)	33	−1.30	6.70	38	−1.20	6.60	−0.01 [−0.48; 0.45]
Self−esteem[35] (Bozzatello 2019)	25	1.90	7.11	29	2.00	5.64	−0.02 [−0.55; 0.52]
Emotional Symptoms[67] (Morris 2017)	33	−5.80	23.90	38	−5.30	18.50	−0.02 [−0.49; 0.44]
Hand mobility[67] (Morris 2017)	33	−26.70	31.90	38	−25.70	35.20	−0.03 [−0.50; 0.44]
Suicide[33] (Blomdahl 2018)	43	0.05	6.02	35	0.31	7.04	−0.04 [−0.49; 0.41]
Hand mobility[55] (Khedekar 2017)	5	−5.51	1.83	5	−5.39	2.98	−0.04 [−1.28; 1.20]
QoL[85] (Roswiyani 2020)	63	0.22	18.93	65	0.95	11.76	−0.05 [−0.39; 0.30]
Cognitive Performance[41] (Ciasca 2018)	31	−3.30	11.90	25	−1.90	10.10	−0.12 [−0.65; 0.40]
Negative affect[67] (Morris 2017)	33	3.20	10.80	38	4.50	9.40	−0.13 [−0.59; 0.34]
Emotional Symptoms[85] (Roswiyani 2020)	63	−5.27	49.47	65	−0.02	25.02	−0.13 [−0.48; 0.21]
Social Adjustment[67] (Morris 2017)	33	−3.40	27.70	38	2.70	34.00	−0.19 [−0.66; 0.27]
Clinical Global Impression[85] (Roswiyani 2020)	63	−2.70	18.18	65	0.46	14.11	−0.19 [−0.54; 0.15]
Hand mobility[55] (Khedekar 2017)	5	−103.12	55.56	5	−90.07	57.28	−0.21 [−1.45; 1.04]
QoL[85] (Roswiyani 2020)	63	−1.49	13.47	65	1.25	8.37	−0.24 [−0.59; 0.10]
Stress[27] (Abbing 2019 b)	18	−1.75	5.96	19	−0.04	7.40	−0.25 [−0.90; 0.40]
Somatic Symptoms[85] (Roswiyani 2020)	63	−7.14	41.26	65	4.23	38.67	−0.28 [−0.63; 0.07]
Clinical Global Impression[35] (Bozzatello 2019)	25	2.20	6.82	29	4.40	7.69	−0.30 [−0.83; 0.24]
Cognitive Performance[63] (Lock 2017)	10	−7.44	6.90	12	−3.33	13.23	−0.36 [−1.21; 0.48]
Positive affect[67] (Morris 2017)	33	−5.40	9.20	38	−1.70	9.90	−0.38 [−0.85; 0.09]
Disruptive behaviour[31] (Beebe 2010)	11	2.18	7.86	11	6.00	7.72	−0.47 [−1.32; 0.38]
QoL[97] (Yamada 2010)	33	−0.10	0.30	30	0.10	0.50	−0.48 [−0.99; 0.02]
Life Satisfaction[97] (Yamada 2010)	33	0.10	4.80	30	3.40	5.80	−0.62 [−1.12; −0.11]
**Random effects model**	**2360**			**2273**			**0.38 [0.26; 0.51]**

Heterogeneity: $I^2 = 71\%$, $\tau^2 = 0.2296$, $p < 0.001$

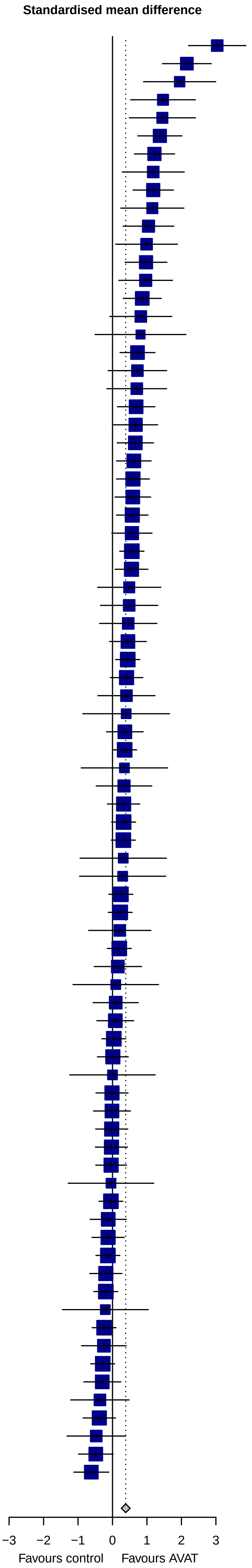

© 2024 Joschko R et al. *JAMA Network Open*.

eFigure 2. Forest plot of the effects of AVAT for studies presenting results as post-test

Outcome	AVAT Total	AVAT Mean	AVAT SD	Control Total	Control Mean	Control SD	Standardised Mean Difference	SMD [95%−CI]
Depression[38] (Ching−Teng 2019)	29	−2.90	2.53	26	−8.62	3.46		1.88 [1.23; 2.52]
Self−esteem[69] (Omizo 1989)	22	64.90	8.40	23	48.10	9.20		1.87 [1.16; 2.58]
Self−esteem[38] (Ching−Teng 2019)	29	27.03	2.58	26	21.54	3.35		1.82 [1.19; 2.46]
Internalising Problems[30] (Bazargan 2016)	14	−27.57	3.75	13	−35.53	5.34		1.68 [0.79; 2.58]
Self−esteem[69] (Omizo 1989)	22	70.20	10.40	23	53.60	9.40		1.65 [0.96; 2.33]
Clinical Global Impression[47] (Haeyen 2018)	28	−67.74	18.39	29	−90.34	17.96		1.23 [0.66; 1.80]
Anxiety[99] (Yu 2016)	33	−17.42	10.42	36	−25.22	7.37		0.86 [0.37; 1.36]
Self−awareness[66] (Montag 2014)	16	26.90	6.50	20	20.60	7.90		0.84 [0.15; 1.53]
Anxiety[99] (Yu 2016)	33	−56.78	11.64	36	−66.11	10.41		0.84 [0.34; 1.33]
Clinical Global Impression[39] (Choi 2020)	19	−2.68	1.42	16	−3.81	1.47		0.77 [0.07; 1.46]
Clinical Global Impression[80] (Rao 2009)	38	−21.10	6.95	38	−26.20	6.57		0.75 [0.28; 1.21]
Anxiety[60] (Laurer 2015)	14	−33.90	9.70	14	−43.40	14.80		0.74 [−0.03; 1.51]
Empathy[66] (Montag 2014)	16	29.30	5.40	20	24.80	6.50		0.73 [0.05; 1.41]
Self−esteem[69] (Omizo 1989)	22	59.10	10.40	23	52.10	9.10		0.70 [0.10; 1.31]
Cognitive Performance[37] (Cetinkaya 2019)	15	24.20	3.60	15	21.40	4.30		0.69 [−0.05; 1.43]
Negative affect[60] (Laurer 2015)	14	−13.60	23.40	14	−36.40	39.90		0.68 [−0.09; 1.44]
Coping[71] (Oster 2006)	20	257.20	28.70	21	233.60	44.10		0.62 [−0.01; 1.25]
QoL[75] (Svensk 2009)	20	78.75	14.68	21	66.67	22.82		0.61 [−0.01; 1.24]
Somatic Symptoms[61] (Lawson 2016)	14	−14.92	10.71	14	−23.38	16.52		0.59 [−0.17; 1.35]
Positive and Negative Symptoms[82] (Richardson 2007)	35	−7.60	3.40	38	−9.90	4.40		0.58 [0.11; 1.04]
Treatment Satisfaction[66] (Montag 2014)	16	16.70	4.50	20	14.10	4.40		0.57 [−0.10; 1.24]
QoL[48] (Hattori 2011)	20	50.50	4.00	19	47.30	6.70		0.57 [−0.07; 1.21]
Self−esteem[69] (Omizo 1989)	22	57.10	10.30	23	51.70	10.40		0.51 [−0.08; 1.11]
Cognitive Performance[73] (Pongan 2017)	27	5.31	7.15	25	2.55	3.23		0.48 [−0.07; 1.04]
Stress[27] (Abbing 2019 b)	19	45.30	40.41	17	29.76	18.99		0.47 [−0.19; 1.14]
QoL[75] (Svensk 2009)	20	−14.76	11.13	21	−20.33	12.23		0.47 [−0.15; 1.09]
Somatic Symptoms[44] (Ellis−Hill 2019)	24	53.80	23.84	22	42.70	23.94		0.46 [−0.13; 1.04]
Depression[39] (Choi 2020)	19	−24.26	8.47	16	−28.00	7.97		0.44 [−0.23; 1.12]
QoL[28] (Abdulah 2018)	30	3.60	0.29	30	3.44	0.42		0.44 [−0.07; 0.95]
Depression[73] (Pongan 2017)	27	−6.82	5.60	25	−9.48	6.39		0.44 [−0.11; 0.99]
Self−awareness[47] (Haeyen 2018)	28	−41.61	4.80	29	−43.52	3.81		0.44 [−0.09; 0.96]
Life Satisfaction[37] (Cetinkaya 2019)	15	10.60	3.00	15	9.10	3.80		0.43 [−0.30; 1.15]
Anxiety[73] (Pongan 2017)	27	−64.11	16.11	25	−72.88	24.71		0.42 [−0.13; 0.97]
Positive and Negative Symptoms[66] (Montag 2014)	24	−27.20	23.50	29	−36.70	22.20		0.41 [−0.14; 0.96]
QoL[75] (Svensk 2009)	20	−16.11	21.77	21	−26.46	29.71		0.39 [−0.23; 1.01]
Psychiatric Symptoms[82] (Richardson 2007)	35	−13.50	6.50	38	−16.50	8.60		0.39 [−0.08; 0.85]
QoL[44] (Ellis−Hill 2019)	24	0.68	0.12	21	0.64	0.08		0.38 [−0.21; 0.97]
Cognitive Performance[48] (Hattori 2011)	20	7.70	5.70	19	6.00	3.00		0.36 [−0.27; 1.00]
Psychiatric Symptoms[76] (Thyme 2009)	20	−0.33	0.41	21	−0.49	0.48		0.35 [−0.27; 0.97]
Stress[61] (Lawson 2016)	14	−67.07	17.76	14	−72.14	9.65		0.34 [−0.40; 1.09]
Social Adjustment[82] (Richardson 2007)	33	117.90	21.20	37	110.70	20.20		0.34 [−0.13; 0.82]
QoL[91] (Sterz 2013)	37	−1.88	0.56	29	−2.08	0.62		0.34 [−0.15; 0.83]
Psychiatric Symptoms[76] (Thyme 2009)	20	−0.59	0.65	21	−0.83	0.77		0.33 [−0.29; 0.95]
Hospitalisation[36] (Broome 2001 children)	18	−1.30	2.30	20	−2.40	4.10		0.32 [−0.32; 0.96]
Empathy[66] (Montag 2014)	16	22.30	4.30	20	20.70	5.50		0.31 [−0.35; 0.97]
QoL[75] (Svensk 2009)	19	38.60	24.88	21	30.16	28.20		0.31 [−0.31; 0.93]
Clinical Global Impression[82] (Richardson 2007)	35	−8.10	4.60	38	−9.60	5.10		0.30 [−0.16; 0.77]
Somatic Symptoms[44] (Ellis−Hill 2019)	25	27.00	38.13	22	17.00	26.03		0.30 [−0.28; 0.87]
Self−esteem[44] (Ellis−Hill 2019)	24	21.60	5.18	22	20.10	5.21		0.28 [−0.30; 0.87]
Clinical Global Impression[66] (Montag 2014)	24	52.40	17.20	29	48.00	14.00		0.28 [−0.26; 0.82]
Sleep[62] (Liu 2018)	20	−33.61	4.71	20	−35.30	7.17		0.27 [−0.35; 0.90]
Anxiety[61] (Lawson 2016)	14	−44.79	5.26	14	−46.15	4.67		0.27 [−0.48; 1.01]
Social Adjustment[44] (Ellis−Hill 2019)	25	72.00	28.25	22	64.20	29.70		0.27 [−0.31; 0.84]
Self−esteem[73] (Pongan 2017)	27	33.75	14.11	25	30.92	5.65		0.26 [−0.29; 0.80]
Psychiatric Symptoms[76] (Thyme 2009)	20	−0.32	0.31	21	−0.40	0.31		0.25 [−0.36; 0.87]
Self−esteem[4] (Yan 2019)	56	30.07	4.91	58	28.91	4.33		0.25 [−0.12; 0.62]
Self−esteem[39] (Choi 2020)	19	24.32	4.15	16	23.44	4.03		0.21 [−0.46; 0.88]
Fatigue[91] (Sterz 2013)	34	−0.87	0.87	29	−1.06	0.97		0.20 [−0.29; 0.70]
Fatigue[91] (Sterz 2013)	34	−1.03	0.91	29	−1.22	0.95		0.20 [−0.29; 0.70]
Self−efficacy/ Locus of Control[91] (Sterz 2013)	37	4.50	0.83	32	4.33	0.92		0.19 [−0.28; 0.67]
Psychiatric Symptoms[76] (Thyme 2009)	20	−0.50	0.57	21	−0.61	0.57		0.19 [−0.42; 0.80]
Fatigue[91] (Sterz 2013)	34	−1.21	1.03	29	−1.41	1.06		0.19 [−0.31; 0.69]
Anxiety[83] (Robinson 2018)	54	−39.69	12.11	52	−42.09	13.31		0.19 [−0.19; 0.57]
Anxiety[44] (Ellis−Hill 2019)	25	−6.30	3.74	22	−7.00	4.13		0.18 [−0.40; 0.75]
QoL[42] (Crawford 2012)	119	0.69	0.27	121	0.64	0.30		0.17 [−0.08; 0.43]
Depression[91] (Sterz 2013)	33	−0.60	0.53	29	−0.69	0.50		0.17 [−0.33; 0.67]
Empathy[57] (Kline 2020)	40	46.90	2.90	43	46.10	6.30		0.16 [−0.27; 0.59]
Social Adjustment[82] (Richardson 2007)	35	−0.90	0.60	37	−1.00	0.70		0.15 [−0.31; 0.61]
Psychiatric Symptoms[82] (Richardson 2007)	34	−0.80	0.50	36	−0.90	0.80		0.15 [−0.32; 0.62]
Social Adjustment[39] (Choi 2020)	19	86.74	11.88	16	85.13	10.93		0.14 [−0.53; 0.80]
Pain[73] (Pongan 2017)	27	−1.22	1.27	25	−1.44	1.89		0.14 [−0.41; 0.68]
PTSD/Trauma[93] (Thyme 2007)	18	−10.61	8.45	21	−11.67	7.64		0.13 [−0.50; 0.76]
QoL[82] (Richardson 2007)	35	4.60	0.70	39	4.50	0.90		0.12 [−0.33; 0.58]
Well−being[44] (Ellis−Hill 2019)	25	44.60	25.65	22	42.00	20.45		0.11 [−0.46; 0.68]
Clinical Global Impression[44] (Ellis−Hill 2019)	25	57.60	19.32	22	55.50	21.49		0.10 [−0.47; 0.67]
Stress[52] (Jouybari 2020)	35	−57.88	27.31	35	−60.20	20.62		0.09 [−0.37; 0.56]
QoL[48] (Hattori 2011)	20	53.40	3.30	19	52.90	6.70		0.09 [−0.53; 0.72]
QoL[75] (Svensk 2009)	16	56.25	26.44	13	53.85	25.60		0.09 [−0.64; 0.82]
Self−efficacy/ Locus of Control[66] (Montag 2014)	16	55.60	13.00	20	54.40	16.50		0.08 [−0.58; 0.74]
Social Adjustment[4] (Yan 2019)	56	−19.48	9.64	58	−20.16	7.81		0.08 [−0.29; 0.44]
Life Satisfaction[4] (Yan 2019)	56	3.05	0.50	58	3.02	0.43		0.06 [−0.30; 0.43]
Stress[87] (Schrade 2011)	16	−68.10	12.50	16	−68.90	14.30		0.06 [−0.64; 0.75]
Well−being[44] (Ellis−Hill 2019)	25	48.40	10.28	22	48.00	8.20		0.04 [−0.53; 0.62]
Social Adjustment[42] (Crawford 2012)	119	−8.30	5.00	121	−8.50	4.90		0.04 [−0.21; 0.29]
QoL[75] (Svensk 2009)	20	−20.42	16.10	21	−21.03	14.58		0.04 [−0.57; 0.65]
QoL[73] (Pongan 2017)	27	−8.89	2.33	25	−9.01	3.86		0.04 [−0.51; 0.58]
QoL[75] (Svensk 2009)	20	81.67	24.57	21	80.95	20.77		0.03 [−0.58; 0.64]
Depression[44] (Ellis−Hill 2019)	25	−6.00	4.18	22	−6.10	3.33		0.03 [−0.55; 0.60]
Anxiety[39] (Choi 2020)	19	−10.74	6.58	16	−10.88	5.01		0.02 [−0.64; 0.69]
Stress[61] (Lawson 2016)	14	−118.00	14.42	14	−118.21	12.39		0.02 [−0.73; 0.76]
Cognitive Performance[73] (Pongan 2017)	27	12.65	8.49	25	12.60	10.03		0.01 [−0.54; 0.55]
Hospitalisation[36] (Broome 2001 children)	18	−1.80	2.90	18	−1.80	3.10		0.00 [−0.65; 0.65]
Depression[48] (Hattori 2011)	20	−3.80	3.00	19	−3.80	1.40		0.00 [−0.63; 0.63]
Stress[87] (Schrade 2011)	16	−73.40	16.00	16	−73.40	16.00		0.00 [−0.69; 0.69]
Cognitive Performance[48] (Hattori 2011)	20	24.40	3.00	19	24.40	2.70		0.00 [−0.63; 0.63]
Stress[61] (Lawson 2016)	14	−89.00	1427.00	14	−84.50	1308.00		−0.00 [−0.74; 0.74]
Positive and Negative Symptoms[53] (Leurent 2014)	116	−62.00	17.30	116	−61.90	19.70		−0.01 [−0.26; 0.25]
Emotional Symptoms[44] (Ellis−Hill 2019)	25	49.30	45.26	22	50.00	42.10		−0.02 [−0.59; 0.56]
Well−being[44] (Ellis−Hill 2019)	25	69.30	20.12	22	69.80	19.51		−0.02 [−0.60; 0.55]
Positive and Negative Symptoms[66] (Montag 2014)	24	−27.60	16.80	29	−27.10	15.50		−0.03 [−0.57; 0.51]
Clinical Global Impression[42] (Crawford 2012)	119	44.90	14.60	121	45.70	14.40		−0.06 [−0.31; 0.20]
Positive and Negative Symptoms[42] (Crawford 2012)	119	−72.70	27.30	121	−71.20	24.60		−0.06 [−0.31; 0.20]
Pain[44] (Ellis−Hill 2019)	25	69.50	27.63	22	71.20	28.32		−0.06 [−0.63; 0.51]
Positive and Negative Symptoms[53] (Leurent 2014)	116	−87.00	31.00	116	−85.20	24.80		−0.06 [−0.32; 0.19]
Pain[73] (Pongan 2017)	27	−3.22	2.50	25	−3.04	2.93		−0.07 [−0.61; 0.48]
Pain[73] (Pongan 2017)	27	−1.93	1.05	25	−1.84	1.38		−0.07 [−0.62; 0.47]
Positive and Negative Symptoms[53] (Leurent 2014)	116	−70.20	25.10	116	−67.90	23.10		−0.10 [−0.35; 0.16]
Well−being[44] (Ellis−Hill 2019)	25	0.76	0.22	22	0.78	0.15		−0.10 [−0.68; 0.47]
Treatment Satisfaction[42] (Crawford 2012)	119	23.60	6.50	121	24.30	6.40		−0.11 [−0.36; 0.15]
Depression[93] (Thyme 2007)	18	−14.44	7.35	21	−13.38	11.03		−0.11 [−0.74; 0.52]
PTSD/Trauma[93] (Thyme 2007)	18	−17.44	9.25	21	−16.43	8.90		−0.11 [−0.74; 0.52]
Executive function[27] (Abbing 2019 b)	24	−62.61	9.95	23	−61.48	9.48		−0.11 [−0.69; 0.46]
Depression[66] (Montag 2014)	24	−6.40	4.50	29	−5.90	3.90		−0.12 [−0.66; 0.42]
Positive and Negative Symptoms[53] (Leurent 2014)	116	−76.60	30.60	116	−73.20	25.80		−0.12 [−0.38; 0.14]
Stress[61] (Lawson 2016)	14	−16.43	1.16	14	−16.29	1.07		−0.12 [−0.86; 0.62]
Apathy[48] (Hattori 2011)	20	−12.70	6.10	19	−11.90	6.70		−0.12 [−0.75; 0.51]
Depression[59] (Lalingkar 2020)	20	−14.80	1.24	20	−14.65	1.09		−0.13 [−0.75; 0.49]
Stress[87] (Schrade 2011)	16	−119.40	16.80	16	−116.40	27.80		−0.13 [−0.82; 0.57]
Pain[73] (Pongan 2017)	27	−4.48	2.78	25	−4.08	3.21		−0.13 [−0.68; 0.41]
Agitation[50] (Hsiaö 2020)	24	−0.41	0.50	8	−0.33	0.19		−0.17 [−0.98; 0.63]
Behavioural Problems[48] (Hattori 2011)	20	−16.80	12.90	19	−14.50	12.70		−0.18 [−0.81; 0.45]
Well−being[42] (Crawford 2012)	119	59.60	20.80	121	64.10	23.70		−0.20 [−0.45; 0.05]
Pain[73] (Pongan 2017)	27	−1.46	0.92	25	−1.24	0.97		−0.23 [−0.78; 0.32]
QoL[75] (Svensk 2009)	20	56.67	28.82	21	63.49	27.70		−0.24 [−0.85; 0.38]
Self−efficacy/ Locus of Control[81] (Regev 2005)	24	46.69	4.07	25	47.76	3.78		−0.27 [−0.83; 0.29]
Cognitive Performance[73] (Pongan 2017)	27	−72.92	30.16	25	−64.95	27.45		−0.27 [−0.82; 0.27]
Self−efficacy/ Locus of Control[81] (Regev 2005)	24	24.19	4.55	25	25.42	4.08		−0.28 [−0.84; 0.28]
Stress[61] (Lawson 2016)	14	−98.29	0.43	14	−98.17	0.39		−0.28 [−1.03; 0.46]
Anxiety[4] (Yan 2019)	56	−3.25	0.92	58	−2.99	0.83		−0.30 [−0.66; 0.07]
Emotional Symptoms[81] (Regev 2005)	24	−35.34	14.35	25	−30.88	10.29		−0.35 [−0.92; 0.21]
Depression[93] (Thyme 2007)	18	−1.53	0.88	21	−1.17	0.92		−0.39 [−1.03; 0.25]
QoL[66] (Montag 2014)	16	202.60	42.20	20	218.30	33.30		−0.41 [−1.07; 0.26]
Executive function[73] (Pongan 2017)	27	13.54	2.78	25	14.58	2.15		−0.41 [−0.96; 0.14]
Hospitalisation[36] (Broome 2001 children)	18	2.80	3.80	22	5.20	5.90		−0.46 [−1.10; 0.17]
Self−esteem[81] (Regev 2005)	24	54.50	10.79	25	59.80	9.83		−0.51 [−1.08; 0.06]
Psychiatric Symptoms[93] (Thyme 2007)	18	−1.10	0.55	21	−0.77	0.60		−0.56 [−1.20; 0.08]
Self−efficacy/ Locus of Control[66] (Montag 2014)	16	56.40	9.30	20	64.60	10.00		−0.83 [−1.51; −0.14]
**Random effects model**	**4279**			**4270**				**0.19 [0.12; 0.26]**

Heterogeneity: $I^2 = 53\%$, $\tau^2 = 0.0777$, $p < 0.001$

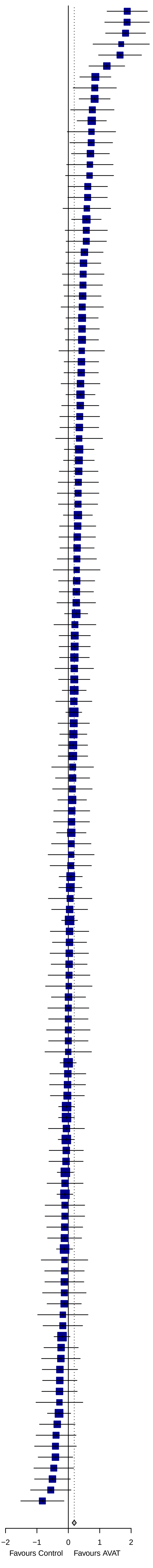

© 2024 Joschko R et al. *JAMA Network Open.*

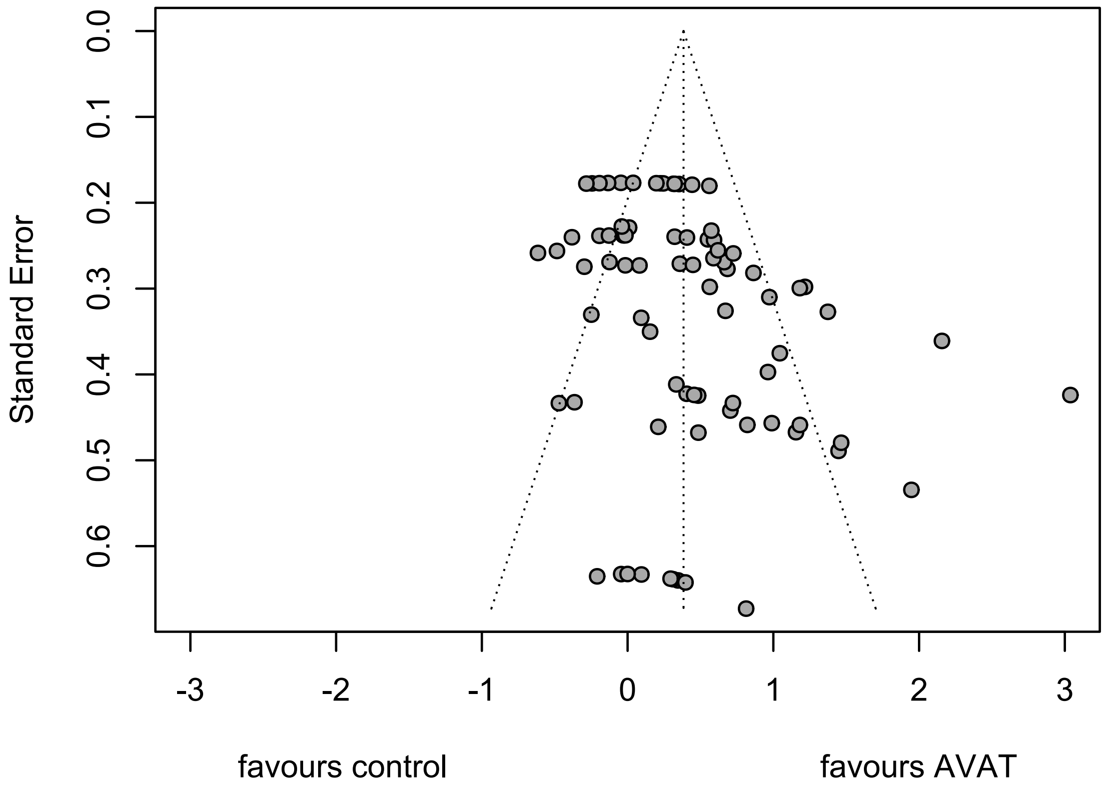

eFigure 3: Funnel plot for change from baseline outcomes.

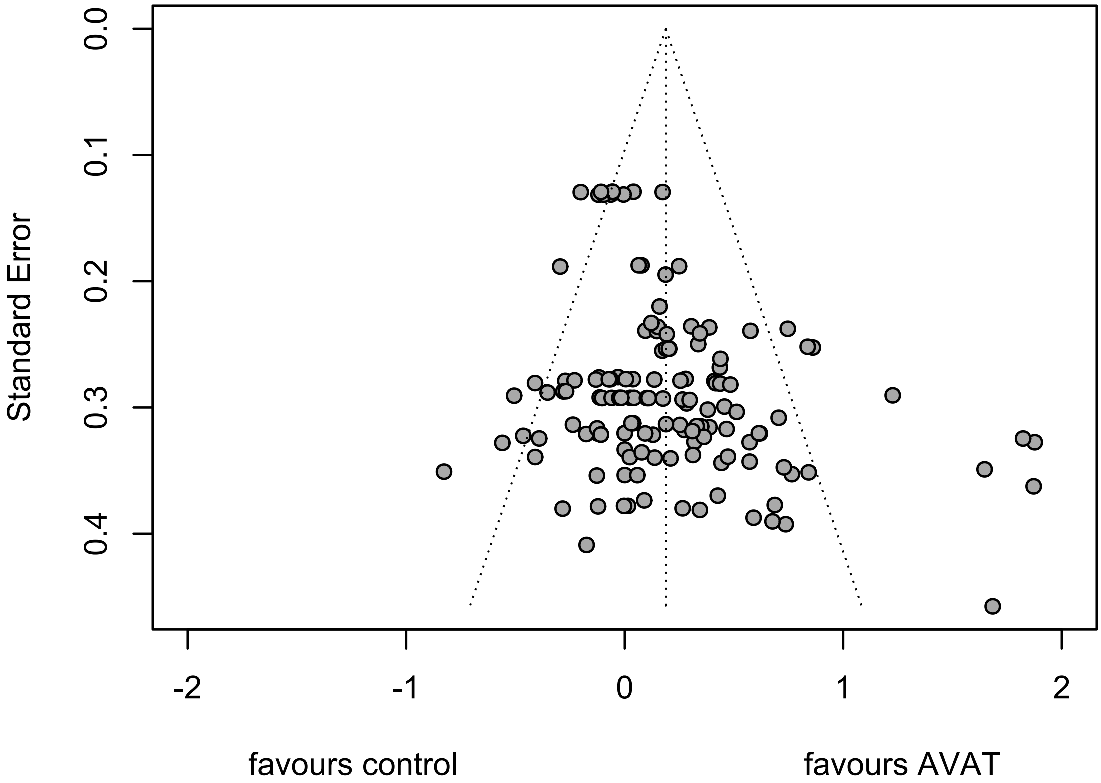

eFigure 4.Funnel plot for post-test outcomes.

© 2024 Joschko R et al. *JAMA Network Open.*

eReferences

1. Abbing A, Baars EW, de Sonneville L, Ponstein AS, Swaab H. The Effectiveness of Art Therapy for Anxiety in Adult Women: A Randomized Controlled Trial. *Frontiers in psychology.* 2019;10(101550902):1203.
2. Abbing A, de Sonneville L, Baars E, Bourne D, Swaab H. Anxiety reduction through art therapy in women. Exploring stress regulation and executive functioning as underlying neurocognitive mechanisms. *PLOS ONE.* 2019;14(12):e0225200-e0225200.
3. Abdulah DM, Abdulla BMO. Effectiveness of group art therapy on quality of life in paediatric patients with cancer: A randomized controlled trial. *Complement Ther Med.* 2018;41:180-185.
4. Barfarazi H, Pourghaznein T, Mohajer S, Mazlom SR, Asgharinekah SM. Evaluating the Effect of Painting Therapy on Happiness in the Elderly. 2018;8(3):17-26.
5. Bazargan Y, Pakdaman S. The Effectiveness of Art Therapy in Reducing Internalizing and Externalizing Problems of Female Adolescents. *Arch Iran Med.* 2016;19(1):51-56.
6. Beebe A, Bender B. A Randomized Trial To Test The Effectiveness Of Art Therapy For Children With Asthma. 2009;123(2 Suppl 1):S64.
7. Beh-Pajooh A, Abdollahi A, Hosseinian S. The effectiveness of painting therapy program for the treatment of externalizing behaviors in children with intellectual disability. *Vulnerable Children and Youth Studies.* 2018;13(3):221-227.
8. Blomdahl C, Guregård S, Rusner M, Wijk H. A manual-based phenomenological art therapy for individuals diagnosed with moderate to severe depression (PATd): A randomized controlled study. *Psychiatric Rehabilitation Journal.* 2018;41(3):169-182.
9. Borchers KK. Do gains made in group art therapy persist? A study with aftercare patients. 1985;23(3):89-91.
10. Bozzatello P, Bellino S, De Marzi G, et al. Effectiveness of psychosocial treatments on symptoms and functional domains in schizophrenia spectrum disorders: a prospective study in a real-world setting. *Disability & Rehabilitation.* 2019;41(23):2799-2806.
11. Broome ME, Maikler V, Kelber S, Bailey P, Lea G. An intervention to increase coping and reduce health care utilization for school-age children and adolescents with sickle cell disease. 2001;12(2):6-14.
12. Çetinkaya F, Aşiret G, Direk F, Özkanlı N. The Effect of Ceramic Painting on the Life Satisfaction and Cognitive Status of Older Adults Residing in a Nursing Home. *Topics in Geriatric Rehabilitation.* 2019;35:108-112.
13. Ching-Teng Y, Ya-Ping Y, Yu-Chia C. Positive effects of art therapy on depression and self-esteem of older adults in nursing homes. *Soc Work Health Care.* 2019;58(3):324-338.
14. Choi H, Jung D-J, Jeon Y-H, Kim MJ. The effects of combining art psychotherapy with pharmacotherapy in treating major depressive disorder: Randomized control study. *The Arts in Psychotherapy.* 2020;70:101689.
15. Ciasca EC, Ferreira RC, Santana CLA, et al. Art therapy as an adjuvant treatment for depression in elderly women: a randomized controlled trial. *Braz J Psychiatry.* 2018;40(3):256-263.
16. Crawford MJ, Killaspy H, Barnes TR, et al. Group art therapy as an adjunctive treatment for people with schizophrenia: a randomised controlled trial (MATISSE). 2012;16(8):iii-iv, 1-76.
17. Leurent B, Killaspy H, Osborn DP, et al. Moderating factors for the effectiveness of group art therapy for schizophrenia: secondary analysis of data from the MATISSE randomised controlled trial. *Social Psychiatry and Psychiatric Epidemiology.* 2014;49(11):1703-1710.
18. Decker KP, Deaver SP, Abbey V, Campbell M, Turpin C. Quantitatively Improved Treatment Outcomes for Combat-Associated PTSD With Adjunctive Art Therapy: randomized Controlled Trial. 2018;35(4):184-194.
19. Ellis-Hill C, Thomas S, Gracey F, et al. HeART of Stroke: randomised controlled, parallel-arm, feasibility study of a community-based arts and health intervention plus usual care compared

with usual care to increase psychological well-being in people following a stroke. 2019;9(3):e021098.

20. Gussak D. Effects of art therapy with prison inmates: A follow-up study. *Arts in Psychotherapy.* 2006;33(3):188-198.
21. Gussak D. The effects of art therapy on male and female inmates: Advancing the research base. *Arts in Psychotherapy.* 2009;36(1):5-12.
22. Haeyen S, van Hooren S, van der Veld W, Hutschemaekers G. Efficacy of Art Therapy in Individuals With Personality Disorders Cluster B/C: A Randomized Controlled Trial. *J Pers Disord.* 2018;32(4):527-542.
23. Hattori H, Hattori C, Hokao C, Mizushima K, Mase T. Controlled study on the cognitive and psychological effect of coloring and drawing in mild Alzheimer's disease patients. *Geriatr Gerontol Int.* 2011;11(4):431-437.
24. Hill KE, Lineweaver TT. Improving the Short-Term Affect of Grieving Children Through Art. 2016;33(2):91-98.
25. Hsiao CY, Chen SL, Hsiao YS, Huang HY, Yeh SH. Effects of Art and Reminiscence Therapy on Agitated Behaviors Among Older Adults With Dementia. *J Nurs Res.* 2020;28(4):e100.
26. Ilali ES, Mokhtary F, Mousavinasab N, Tirgari AH. Impact of Art-Based Life Review on Depression Symptoms Among Older Adults. 2018;35(3):148-155.
27. Jouybari L, Abbariki E, Jebeli M, et al. Comparison of the effect of narrative writing and art therapy on maternal stress in neonatal intensive care settings. 2018.
28. Katz C, Barnetz Z, Hershkowitz I. The effect of drawing on children's experiences of investigations following alleged child abuse. *Child Abuse Negl.* 2014;38(5):858-867.
29. Khedekar S, Shimpi AP, Shyam A, Sancheti P. Use of Art as Therapeutic Intervention for Enhancement of Hand Function in Patients with Rheumatoid Arthritis: A Pilot Study. *Indian Journal of Rheumatology.* 2017;12(2).
30. Kim SK. A randomized, controlled study of the effects of art therapy on older Korean-Americans' healthy aging. *The Arts in Psychotherapy.* 2013;40(1):158-164.
31. Kline JA, Vanryzin K, Davis JC, et al. Randomized Trial of Therapy Dogs Versus Deliberative Coloring (Art Therapy) to Reduce Stress in Emergency Medicine Providers. *Academic Emergency Medicine.* 2020;27(4):266-275.
32. Kopytin A, Lebedev A. Humor, Self-Attitude, Emotions, and Cognitions in Group Art Therapy With War Veterans. 2013;30(1):20-29.
33. Lalingkar RA, Yadav TS, Gijare SS. Effect of Recreational Therapy on Attention and Depression in School Children with Learning Difficulties. *Indian Journal of Public Health Research & Development.* 2020;11(3):573-578.
34. Laurer M, van der Vennet R. Effect of Art Production on Negative Mood and Anxiety for Adults in Treatment for Substance Abuse. 2015;32(4):177-183.
35. Lawson LM, Williams P, Glennon C, et al. Effect of Art Making on Cancer-Related Symptoms of Blood and Marrow Transplantation Recipients. 2012;39(4):E353.
36. Liu C. *Examining the effectiveness of solution-focused art therapy (SF-AT) for sleep problems of children with traumatic experience* [Dissertation], ProQuest Information & Learning; 2018.
37. Lock J, Fitzpatrick KK, Agras WS, Weinbach N, Jo B. Feasibility Study Combining Art Therapy or Cognitive Remediation Therapy with Family-based Treatment for Adolescent Anorexia Nervosa. *European Eating Disorders Review.* 2018;26(1):62-68.
38. Masika GM, Yu DSF, Li PWC. Can Visual Art Therapy Be Implemented With Illiterate Older Adults With Mild Cognitive Impairment? A Pilot Mixed-Method Randomized Controlled Trial. 2021;34(1):76-86.
39. McCaffrey R. The effect of healing gardens and art therapy on older adults with mild to moderate depression. *Holistic Nursing Practice.* 2007;21(2):79-84.
40. Montag C, Haase L, Seidel D, et al. A pilot RCT of psychodynamic group art therapy for patients in acute psychotic episodes: feasibility, impact on symptoms and mentalising capacity. *PLoS One.* 2014;9(11):e112348.

© 2024 Joschko R et al. *JAMA Network Open.*

41. Morris JH, Kelly C, Joice S, et al. Art participation for psychosocial wellbeing during stroke rehabilitation: a feasibility randomised controlled trial. *Disability and Rehabilitation.* 2019;41(1):9-18.
42. Narme P, Tonini A, Khatir F, Schiaratura L, Clément S, Samson S. Thérapies non médicamenteuses dans la maladie d'Alzheimer: Comparison d'ateliers musicaux et non musicaux = Non pharmacological treatment for Alzheimer's disease: Comparison between musical and non-musical interventions. *Gériatrie et Psychologie Neuropsychiatrie du Vieillissement.* 2012;10(2):215-215.
43. Omizo MM, Omizo SA. Art Activities to Improve Self-Esteem Among Native Hawaiian Children. *The Journal of Humanistic Education and Development.* 1989;27(4):167-176.
44. Öster I, Svensk A-C, Magnusson E, et al. Art therapy improves coping resources: A randomized, controlled study among women with breast cancer. *Palliative and Supportive Care.* 2006;4(1):57-64.
45. Öster I, Magnusson E, Thyme KE, Lindh J, Astrom S. Art therapy for women with breast cancer: the therapeutic consequences of boundary strengthening. 2007;34(3):277-288.
46. Öster I, Tavelin B, Thyme K, et al. Art therapy during radiotherapy - A five-year follow-up study with women diagnosed with breast cancer. *The Arts in Psychotherapy.* 2014;41.
47. Thyme KE, Sundin Ec Fau - Wiberg B, Wiberg B Fau - Oster I, Oster I Fau - Aström S, Aström S Fau - Lindh J, Lindh J. Individual brief art therapy can be helpful for women with breast cancer: a randomized controlled clinical study. 2009(1478-9523 (Electronic)).
48. Svensk A-C, Öster I, Thyme KE, et al. Art therapy improves experienced quality of life among women undergoing treatment for breast cancer: a randomized controlled study. *European Journal of Cancer Care.* 2009;18(1):69-77.
49. Pongan E, Tillmann B, Leveque Y, et al. Can Musical or Painting Interventions Improve Chronic Pain, Mood, Quality of Life, and Cognition in Patients with Mild Alzheimer's Disease? Evidence from a Randomized Controlled Trial. 2017;60(2):663-677.
50. Qin Y. Effect of Music Therapy Intervention on Physical Functions and Mental Health of Patients with Ankylosing Spondylitis. *Psychiatr Danub.* 2020;32(3-4):403-410.
51. Agbaria Q. Acquiring social and cognitive skills in an intervention for Arab parents of children with intellectual developmental disability accompanied by behavioral conditions. *Child & Family Social Work.* 2020;25(S1):73-82.
52. Rajendran N, Mitra TP, Shahrestani S, Coggins A. Randomized Controlled Trial of Adult Therapeutic Coloring for the Management of Significant Anxiety in the Emergency Department. *Acad Emerg Med.* 2020;27(2):92-99.
53. Ramirez K, Haen C, Cruz RF. Investigating impact: The effects of school-based art therapy on adolescent boys living in poverty. *The Arts in Psychotherapy.* 2020;71.
54. Rao D, Nainis N, Williams L, Langner D, Eisin A, Paice J. Art therapy for relief of symptoms associated with HIV/AIDS. *AIDS Care.* 2009;21(1):64-69.
55. Regev D, Guttmann J. The psychological benefits of artwork: the case of children with learning disorders. 2005;32(4):302-312.
56. Richardson P, Jones K, Evans C, Stevens P, Rowe A. Exploratory RCT of art therapy as an adjunctive treatment in schizophrenia. 2007;16(4):483-491.
57. Robinson EM, Baker R, Hossain MM. Randomized Trial Evaluating the Effectiveness of Coloring on Decreasing Anxiety Among Parents in a Pediatric Surgical Waiting Area. *J Pediatr Nurs.* 2018;41:80-83.
58. Rosal ML. Comparative group art therapy research to evaluate changes in locus of control in behavior disordered children. *The Arts in Psychotherapy.* 1993;20(3):231-241.
59. Roswiyani R, Hiew CH, Witteman CLM, Satiadarma MP, Spijker J. Art activities and qigong exercise for the well-being of older adults in nursing homes in Indonesia: a randomized controlled trial. *Aging & mental health.* 2020;24(10):1569-1578.
60. Rusted J, Sheppard L, Waller D. A Multi-centre Randomized Control Group Trial on the Use of Art Therapy for Older People with Dementia. *Group Analysis.* 2006;39(4):517-536.

© 2024 Joschko R et al. *JAMA Network Open.*

61. Schrade C, Tronsky L, Kaiser DH. Physiological effects of mandala making in adults with intellectual disability. *The Arts in Psychotherapy.* 2011;38(2):109-113.
62. Schreier H, Ladakakos C, Morabito D, Chapman L, Knudson MM. Posttraumatic stress symptoms in children after mild to moderate pediatric trauma: a longitudinal examination of symptom prevalence, correlates, and parent-child symptom reporting. *J Trauma.* 2005;58(2):353-363.
63. Siegel J, Iida H, Rachlin K, Yount G. Expressive Arts Therapy with Hospitalized Children: A Pilot Study of Co-Creating Healing Sock Creatures(c). *J Pediatr Nurs.* 2016;31(1):92-98.
64. Yount G, Rachlin K, Siegel J. Expressive arts therapy for hospitalized children: a pilot study measuring cortisol levels. *Pediatr Rep.* 2013;5(2):28-30.
65. Sommer F, Obenaus K, Engelmann U. Creative-dynamic image synthesis: a useful addition to the treatment options for impotence. 2001;13(5):268-274; discussion 275.
66. Sterz C, Heimes S, Blessing T, Flachenecker P. Creative arts therapy improves quality of life in MS - Results of a randomized controlled trial during inpatient rehabilitation. 2013;19(3):176-182.
67. Sukru F, Ozturk M, Kilic O, Guneytepe S, Ucok A. The impact of a six-month interpersonal group psychotherapy on functionality of patients with schizophrenia in a community mental health center. 2018.
68. Thyme KE, Sundin EC, Stahlberg G, Lindstrom B, Eklof H, Wiberg B. The outcome of short-term psychodynamic art therapy compared to short-term psychodynamic verbal therapy for depressed women. *Psychoanalytic Psychotherapy.* 2007;21(3):250-264.
69. Tibbetts TJ, Stone B. Short-term art therapy with seriously emotionally disturbed adolescents. *The Arts in Psychotherapy.* 1990;17(2):139-146.
70. Wahlbeck H, Kvist LJ, Landgren K. Art Therapy and Counseling for Fear of Childbirth: A Randomized Controlled Trial. *Art Therapy.* 2020;37(3):123-130.
71. Wu C-J, Lee S-Y. After the interventionafter the intervention an evaluation and analysis of visual art therapy in the treatment of PTSD. 2020.
72. Yamada T, Kawamata H, Kobayashi N, Kielhofner G, Taylor RR. A Randomised Clinical Trial of a Wellness Programme for Healthy Older People. *British Journal of Occupational Therapy.* 2010;73(11):540-548.
73. Yan H, Chen J, Huang J. School bullying among left-behind children: the efficacy of art therapy on reducing bullying victimization. 2019;10(FEB).
74. Yu YZ, Ming CY, Yue M, Li JH, Ling L. House–Tree–Person drawing therapy as an intervention for prisoners' prerelease anxiety. *Social Behavior and Personality: An International Journal.* 2016;44(6):987-1004.
75. Bacha A. Impact of art therapy on brain connectivity in recent post-stroke aphasia (ART-CONNECT). 2019.
76. Beauchet O, Cooper-Brown L, Hayashi Y, Galery K, Vilcocq C, Bastien T. Effects of "thursdays at the Museum" at the Montreal Museum of Fine Arts on the mental and physical health of older community dwellers: The art-health randomized clinical trial protocol. *Trials.* 2020;21(1):709.
77. Chan CKP, Lo TLT, Wan AHY, Leung PPY, Pang MYC, Ho RTH. A randomised controlled trial of expressive arts-based intervention for young stroke survivors. *BMC Complementary Medicine and Therapies.* 2021;21(1):7.
78. Drks. Art therapy as a preventive or interventional treatment for geriatric patients. In:2020.
79. Drks. Art therapy to regulate hypertension - a quantitative, controlled study. In:2018.
80. Singler K. Kunsttherapie mit älteren Menschen in Nürnberg DUERER. In: Klinikum NürnbergKlinik für Innere Medizin II, Schwerpunkt für Geriatrie.
81. Waller C. Psycho-biologische Behandlungseffekte traumafokussierter Maltherapie bei Patientinnen mit psychischen und physischen Traumafolgereaktionen MALT! In: Klinikum Nürnberg; 2020.
82. Nan J. The effects of art therapy with clay on emotion regulation, stress, and hormone levels in secondary school students. In: Hong Kong Baptist University; 2020.

© 2024 Joschko R et al. *JAMA Network Open.*

83. Joly F, Pasquier D, Hanzen C, et al. Impact of art therapy (AT) on fatigue and quality of life (QoL) during adjuvant external beam irradiation (EBI) in breast cancer patients (pts): a randomized trial. 2016;27:vi499-.
84. Lee S, Kim M, Seo JS, Lee K, Jon D, Bahk W. T103. THE EFFECTS OF GROUP ARTS THERAPY BASED ON EMOTION MANAGEMENT TRAINING ON THE EMOTIONAL EXPRESSION, ALEXITHYMIA, DEPRESSION AND QUALITY OF LIFE IN PATIENTS WITH SCHIZOPHRENIA...2019 Congress of the Schizophrenia International Research Society, 10-1. 2019;45:S243.
85. Matus P. Impact of waiting room art intervention on pediatric patients' anxiety and cooperation with physician. *Journal of Investigative Medicine.* 2020;68(Supplement 1).
86. Naveed A, Masood S. A comparison of group art therapy in decreasing the depression level of leukamia paediatric patients. *Pediatric Blood and Cancer.* 2020;67(SUPPL 4).
87. Paillard EB. Application of Art Therapy in Oncology: Evaluation of the Symptomatic of Patients Suffering From a Cancerous Disease (ARONCO). In: Centre Hospitalier de Perigueux; 2020.
88. Nahleh Z. Assessing the Benefit of Art & Music Therapy on Quality of Life in Patients With Breast Cancer. In: Cleveland Clinic; 2020.
89. Clavien P-A. The Effects of Perioperative Painting Art Therapy (LOM® Solution Centered Art Therapy) in Surgical Patients. In: University of Zurich; 2020.
90. Ho AHY. ARTISAN 2.0: deconstructing the Integrative Efficacy of a Multimodal Art-based Intervention. In: Nanyang Technological University, National Museum of Singapore; 2020.
91. Suleman SK, Atrushi A, Enskar K. Effectiveness of art-based distraction in reducing pain and anxiety of hospitalized children during cannulation procedure: A randomized controlled trial. *Belitung Nurs J.* 2022;8(3):213-221.
92. Pieper C, Schroer S, Spanier H, Cohen S, Russ H, Baer U. The UPGRADE study protocol: creative strengthening groups as an innovative preventive intervention for healthcare workers. 2020;70(11):475-480.
93. Gohier B. Impact of Art Therapy on Alexithymia in People With Alcohol Use Disorder. In. *https://clinicaltrials.gov/show/NCT04206930*: University Hospital Angers; 2019.
94. Page MJ, McKenzie JE, Bossuyt PM, et al. The PRISMA 2020 statement: an updated guideline for reporting systematic reviews. *Systematic Reviews.* 2021;10(1).

© 2024 Joschko R et al. *JAMA Network Open.*
