# Supplementary material for: Active Visual Art Therapy and Health Outcomes: A Systematic Review and Meta-Analysis
Source: JAMA Netw Open. 2024 Sep 12;7(9):e2428709. doi: 10.1001/jamanetworkopen.2024.28709 (PMC11393726; doi:10.1001/jamanetworkopen.2024.28709)
Supplement: Supplement 3. — Data Sharing Statement [file jamanetwopen-e2428709-s003.pdf]

Data Sharing Statement

Joschko. Active Visual Art Therapy and Health Outcomes. *JAMA Netw Open*. Published September 12, 2024. doi:10.1001/jamanetworkopen.2024.28709

Data
Data available: No

Additional Information
Explanation for why data not available: The datasets used during this review are available from the corresponding author upon reasonable request.
